# Quantitative Control of Protein *S*-Palmitoylation Regulates Meiotic Entry in Fission Yeast

**DOI:** 10.1371/journal.pbio.1001597

**Published:** 2013-07-02

**Authors:** Mingzi M. Zhang, Pei-Yun Jenny Wu, Felice D. Kelly, Paul Nurse, Howard C. Hang

**Affiliations:** 1 Laboratory of Chemical Biology and Microbial Pathogenesis, The Rockefeller University, New York, New York, United States of America; 2 Laboratory of Yeast Genetics and Cell Biology, The Rockefeller University, New York, New York, United States of America; Wayne State University, United States of America

## Abstract

Protein *S*-palmitoylation, a lipid modification mediated by members of the palmitoyltransferase family, serves as an important membrane-targeting mechanism in eukaryotes. Although changes in palmitoyltransferase expression are associated with various physiological and disease states, how these changes affect global protein palmitoylation and cellular function remains unknown. Using a bioorthogonal chemical reporter and labeling strategy to identify and analyze multiple cognate substrates of a single Erf2 palmitoyltransferase, we demonstrate that control of Erf2 activity levels underlies the differential modification of key substrates such as the Rho3 GTPase in vegetative and meiotic cells. We show further that modulation of Erf2 activity levels drives changes in the palmitoylome as cells enter meiosis and affects meiotic entry. Disruption of Erf2 function delays meiotic entry, while increasing Erf2 palmitoyltransferase activity triggers aberrant meiosis in sensitized cells. Erf2-induced meiosis requires the function of the Rho3 GTPase, which is regulated by its palmitoylation state. We propose that control of palmitoyltransferase activity levels provides a fundamental mechanism for modulating palmitoylomes and cellular functions.

## Abstract 2

Transcriptional control of DHHC palmitoyltransferase activity levels shapes cellular palmitoylation patterns and influences meiotic entry in fission yeast.

## Author Summary

Palmitoylation is a fatty acid modification, which increases the hydrophobicity of proteins, known to control the membrane trafficking and function of many proteins important for cellular homeostasis and disease. Being a reversible process, palmitoylation is proposed to be a major cellular regulator. While changes in global protein palmitoylation patterns are often associated with different physiological states, the mechanisms that regulate this posttranslational modification and their functional consequences have been elusive. Using improved methods for protein palmitoylation analysis in the genetically tractable fission yeast *Schizosaccharomyces pombe*, we discovered that the global protein palmitoylation pattern is significantly altered and correlates with entry into meiosis, a major developmental transition. We further showed that the precise level of a palmitoylating enzyme is important to shape the subset of lipid-modified proteins and influence the start of this cellular transition. As palmitoylated proteins are involved in eukaryotic cellular pathways, our results provide a crucial insight into how the quantitative control of palmitoylating enzymes may impact human physiology and disease.

## Introduction

In eukaryotic cells, posttranslational protein *S*-palmitoylation (hereafter referred to as palmitoylation) regulates protein-membrane associations as well as protein targeting and trafficking. The covalent addition of palmitic acid to cysteine residues is vital for the function of key signaling factors in diverse cellular processes including immune and neuronal signaling [1]–[3]. With its potential for reversibility [4], which is unique among the characterized lipid modifications, palmitoylation allows both spatial and temporal control of protein function.

Given its versatility, palmitoylation is poised to be a major cellular regulator akin to phosphorylation [5]. Indeed, growing evidence suggests that protein palmitoylation is actively regulated. For example, receptor activity-regulated palmitate turnover of PSD-95 modulates synaptic strength and plasticity in postsynaptic neurons by mediating receptor clustering [6],[7]. Other extracellular signals have also been shown to alter the palmitoylation state or palmitate turnover rate of individual proteins in different cell types [8]–[11]. Additionally, a broader level of control is suggested by global changes in palmitoylomes associated with specific physiological states of the cell or organism [12],[13]. While protein interactions that affect substrate accessibility have been shown to affect the palmitoylation state of specific substrates [14], the biochemical and genetic mechanisms that globally shape cellular palmitoylomes in response to physiological cues have remained elusive.

Protein palmitoylation is catalyzed by the Asp-His-His-Cys (DHHC)-containing palmitoyltransferases (hereafter referred to as palmitoyltransferases) [15]–[17]. One of the first palmitoyltransferases discovered, ERF2 (Effectors of Ras Function 2), was identified from a budding yeast screen designed to uncover cellular regulators of Ras palmitoylation [16]. Together, ERF2 (YLR246W) and its accessory protein ERF4 (YOL110W) form a functional palmitoyltransferase complex that catalyzes palmitate transfer to Ras [17]–[19]. Members of this palmitoyltransferase family are responsible for most protein palmitoylation events [20], and orthologs of these integral membrane enzymes with their characteristic DHHC motif that is required for palmitoyltransferase activity have been found in all examined eukaryotic genomes. In addition to having both distinct and overlapping substrate specificities [20]–[22], palmitoyltransferases exhibit varying intracellular localization and tissue-specific expression [23],[24]. Misregulated expression of specific palmitoyltransferases in mammals is associated with various developmental defects [25],[26], neurological disorders [27],[28], and cancers [29],[30]. Yet despite their central roles in transferring palmitate to proteins, to date there is little evidence for regulation of palmitoyltransferases, and the roles of these enzymes in shaping global protein palmitoylation in physiological contexts remain largely unexplored.

To investigate how palmitoyltransferase regulation affects global protein palmitoylation in a physiological context, we integrated genetic and chemical tools in the fission yeast *Schizosaccharomyces pombe* and show that quantitative control of protein palmitoylation by varying Erf2 palmitoyltransferase activity levels shapes the palmitoylome during meiosis. We further demonstrate that palmitoyltransferase level-mediated changes in substrate palmitoylation affect meiotic entry in fission yeast cells, highlighting the physiological relevance of this regulatory mechanism for global protein palmitoylation.

## Results

### Changes in Global Protein Palmitoylation Are Associated with Meiosis

Fission yeast cells normally proliferate in the haploid state, but when nutrients become limiting, cells may enter an alternate meiotic differentiation pathway: cells of opposite mating types conjugate to form a diploid zygote, which replicates its genome and then undergoes two successive nuclear divisions to yield four haploid nuclei that mature into spores (Figure S1A). We took advantage of a well-characterized system in fission yeast for inducing synchronous meiosis that uses a temperature-sensitive allele of the Pat1 meiotic repressor (SPBC19C2.05), *pat1-114* [31]–[33]. Diploid cells harboring this mutation can be induced to undergo meiosis in a timely and predictable manner by shifting the cultures from a permissive to restrictive temperature, resulting in the formation of viable spores (Figures S1B and 1A,B).

**Figure 1:**
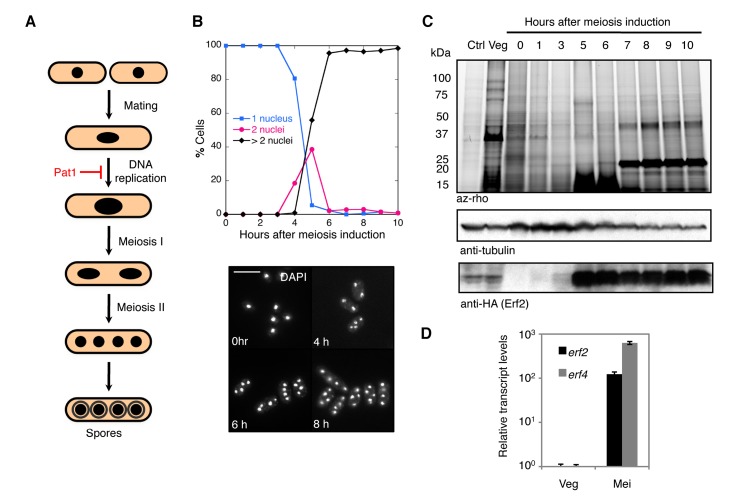
Use of chemical reporter strategy reveals changes in global protein palmitoylation during meiosis in fission yeast. (A) Schematic representation of fission yeast sexual differentiation. Haploid cells conjugate to form a diploid zygote, which undergoes meiosis to yield four haploid nuclei that matures into spores. Pat1 kinase is a general repressor of meiosis. For more details on *S. pombe* meiosis, see Figure S1. (B) Percentage of cells with 1, 2, or >2 nuclei were determined by counting ≥200 DAPI-stained cells of the indicated strains at hourly intervals after meiotic induction (top). Representative DAPI images of cells at indicated times (bottom). Scale bars, 10 µm. Synchronous meiosis in diploid *pat1-114/pat1-114* cells was induced by shifting nitrogen-starved cultures to restrictive temperature (see Materials and Methods). Indicated times refer to the elapsed time after temperature shift. (C) Fluorescence profile of palmitoylated substrates in *erf2-HA_3_/erf2-HA_3_ pat1-114/pat1-114* and *erf2Δ/erf2Δ pat1-114/pat1-114* cells undergoing synchronized meiosis (top panel). At the indicated times after meiotic induction, aliquots of the culture were pulse-labeled with alk-16. Western blots were probed for tubulin and HA (middle and bottom panels). (D) qPCR analysis of *erf2* and *erf4* transcripts, normalized to *act1* mRNA levels, in vegetative (Veg) and meiotic (Mei, 8 h after meiotic induction) *pat1-114/pat1-11*4 cells. Data represent mean values of three biological repeats ± SD.

To analyze global changes in the palmitoylome during meiosis, we employed an alkyne-functionalized palmitate chemical reporter, alk-16, which together with bioorthogonal labeling offers significantly improved fluorescent detection of the modification compared to conventional radiolabeled lipids (Figure S2A–D) [4],[8],[34],[35]. Using this system in diploid cells undergoing synchronous meiosis, we observed a distinct meiotic palmitoylome with a prominent band at approximately 23 kDa, which was absent in vegetative cells (Figure 1C). This prompted us to focus on identifying the enzymes responsible for these modifications. Given that appearance of the meiotic palmitoylome followed the striking increase in Erf2 protein levels (Figure 1C), which reflects the reported *erf2* transcription profile during meiosis [36],[37], we postulated that the DHHC-containing Erf2 protein (SPBC3H7.09) plays a critical role in orchestrating meiosis-specific protein palmitoylation.

### The Erf2 Palmitoyltransferase Drives Changes in the Palmitoylome During Meiosis

**Figure 2:**
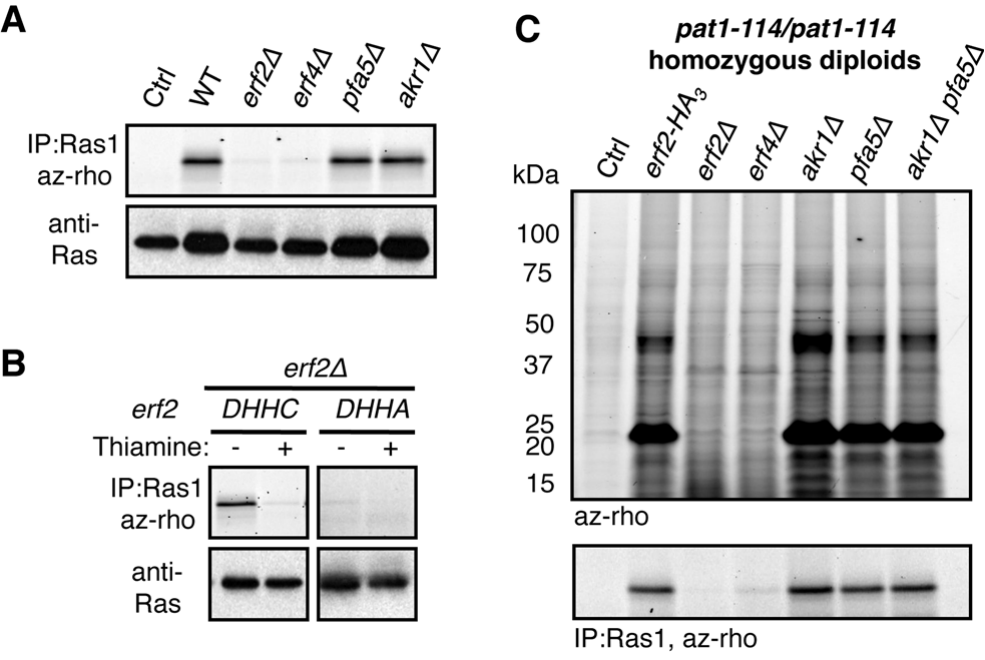
The Erf2–Erf4 palmitoyltransferase drives major changes in the meiotic palmitoylome during meiosis. (A–B) Alk-16-associated fluorescence of immunopurified Ras1 from vegetatively growing cells with the indicated palmitoyltransferase deletions (top panels). Western blots are probed for Ras1 (bottom panels). Wild-type (*DHHC*) or catalytically inactive (*DHHA*) Erf2 was expressed from a thiamine-repressible promoter in *erf2Δ* cells (B). (C) Fluorescence detection of palmitoylated substrates (top panel) and Ras1 palmitoylation (bottom panel) in homozygous diploid *pat1-114/pat1-114* cells with the indicated palmitoyltransferase deletions 8 h after meiotic induction. Ctrl, DMSO control; Veg, vegetative cells.

Of the five open reading frames in the fission yeast genome that encode for proteins containing the DHHC motif, a characteristic feature of palmitoyltransferases (Figure S3A), we focused on Erf2 whose strongly elevated expression during meiosis precedes the appearance of the meiotic palmitoylome (Figures 1D and S3B) [36],[37]. By in-gel fluorescence, we showed that Erf2 and its cofactor Erf4 (SPAC3F10.07c), and not the other DHHC-containing proteins examined, are specifically required for the incorporation of alk-16 onto Ras1 (SPAC17H9.09c) (Figure 2A), the only reported palmitoylated protein in *S. pombe* whose modification is important for pheromone signaling [38]. Incorporation of alk-16 directly requires Erf2 palmitoyltransferase activity, as the Ras1 palmitoylation defect in *erf2Δ* cells is rescued by re-introduction of Erf2, but not a catalytically inactive DHHC→DHHA mutant (Figure 2B) [39]. Collectively, these experiments demonstrate that Erf2 and Erf4 are functional orthologs of the *S. cerevisiae* ERF2 and ERF4 palmitoyltransferase subunits, respectively [17]–[19], and that this method can be used to profile Erf2-Erf4 substrates in fission yeast.

To determine whether Erf2-Erf4 palmitoyltransferase function has an impact on the meiotic palmitoylome, we profiled palmitoylated proteins in mutant diploid cells undergoing synchronous meiosis. The distinct meiotic palmitoylome requires Erf2 activity, as modification of those prominent substrates was not observed in either meiotic *erf2Δ* or *erf4Δ* cells (Figure 2C). Ras1 was also palmitoylated in an Erf2-Erf4-dependent manner in this context (Figure 2C), suggesting a role for Ras1 in meiosis [40]. Since salient features of the meiotic palmitoylome were still observed in the absence of two other putative palmitoyltransferases examined, we concluded that Erf2-Erf4 is the primary palmitoyltransferase driving changes in protein palmitoylation during meiosis.

This system, in which palmitoyltransferase regulation is linked to a highly coordinated biological process, provides an excellent model for studying how control of palmitoyltransferase expression affects global protein palmitoylation and its functional consequences.

### Differential Palmitoylation of Erf2 Substrates in Distinct Cellular States

**Figure 3:**
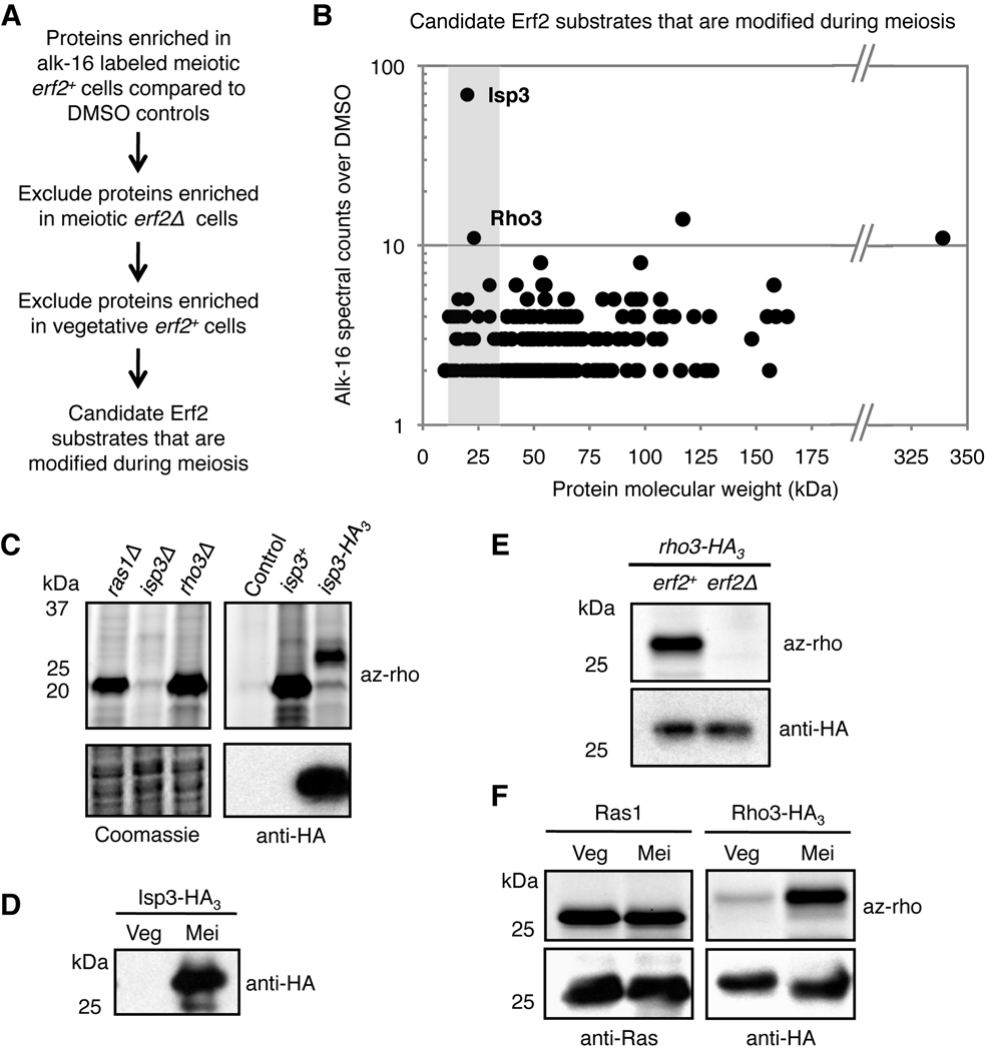
Erf2 substrates are differentially modified in vegetative and meiotic cells. (A) Filter criteria for candidate substrates that are palmitoylated by Erf2 during meiosis. (B) Each of the 238 candidates obtained is represented as a data point reflecting its molecular weight and enrichment (net spectral counts) in alk-16 over DMSO labeled *erf2^+^* meiotic cells. Isp3 and Rho3 are the top two candidates with molecular weights ∼23 kDa (shaded). (C) Fluorescence profiles of meiotic cells with indicated gene deletions or expressing endogenous or tagged Isp3 (top panels). Western blot is probed for HA (bottom panels). Ctrl, DMSO control. (D) Isp3-HA_3_ expression as determined by anti-HA blot of lysates from cells in distinct cellular states. Veg, vegetative cells. Mei, meiotic cells. (E) Rho3-HA_3_ palmitoylation in meiotic *erf2^+^* and *erf2Δ* cells (top panel). Western blot is probed for HA (bottom panel). (F) Ras1 and Rho3 palmitoylation states in vegetative and meiotic cells (top panels). Western blots were probed for Ras1 and HA (bottom panels). Synchronous meiosis in indicated diploid *pat1-114/pat1-114* cells was induced by shifting nitrogen-starved cultures to restrictive temperature (see Materials and Methods). Meiotic cells refer to cells 8 h after the temperature shift.

To study how control of palmitoyltransferase expression affects palmitoylation of its substrates, we sought to identify the Erf2 substrates that are preferentially palmitoylated during meiosis by comparative proteomics. We affinity purified alk-16-labeled proteins in meiotic *erf2^+^*, vegetative *erf2^+^*, and meiotic *erf2Δ* cells using a cleavable azido-biotin affinity tag (Figure S4A–C) [41]. Recovered proteins were subsequently identified by gel-based proteomics, and those with ≥2-fold spectral counts in alk-16 samples compared to the corresponding DMSO controls were further analyzed. Proteins enriched in meiotic *erf2^+^* cells were subjected to two filter criteria, excluding those also enriched in (1) meiotic *erf2Δ* cells and (2) vegetative *erf2^+^* cells (Figure 3A). Notably, Ras1 was excluded because it was equally recovered in both meiotic and vegetative *erf2^+^* cells, consistent with our biochemical analyses (Figure 2B,C). Of the 238 remaining candidates (Table S2), we focused on Isp3 (SPAC1F8.05) and Rho3 (SPAC23C4.08) as they were the most highly enriched proteins with molecular weights matching the prominent ∼23 kDa band we previously observed (Figures 3B and S4D). Isp3, an abundant protein involved in spore formation with meiosis-specific expression (Figure 3D) [42],[43], was the most heavily modified species: the ∼23 kDa band was lost in *isp3Δ* cells, and HA_3_-tagging of Isp3 reduced the electrophoretic mobility of the major fluorescent band (Figure 3C). We further confirmed that Rho3, a Rho GTPase involved in polarized growth in vegetative cells [44],[45], is also an Erf2 substrate (Figure 3E) that is differentially modified between meiotic and vegetative cells (Figure 3F). These results suggest a novel mechanism by which changes in the palmitoylome can be mediated by the regulation of a single palmitoyltransferase activity. Given that Erf2 expression is low in vegetative cells and high in meiotic cells (Figure 1D), we reasoned that the differential modification of Rho3 and Ras1 could be a consequence of palmitoyltransferase levels.

### Quantitative Control of Protein Palmitoylation by Varying Palmitoyltransferase Levels

**Figure 4:**
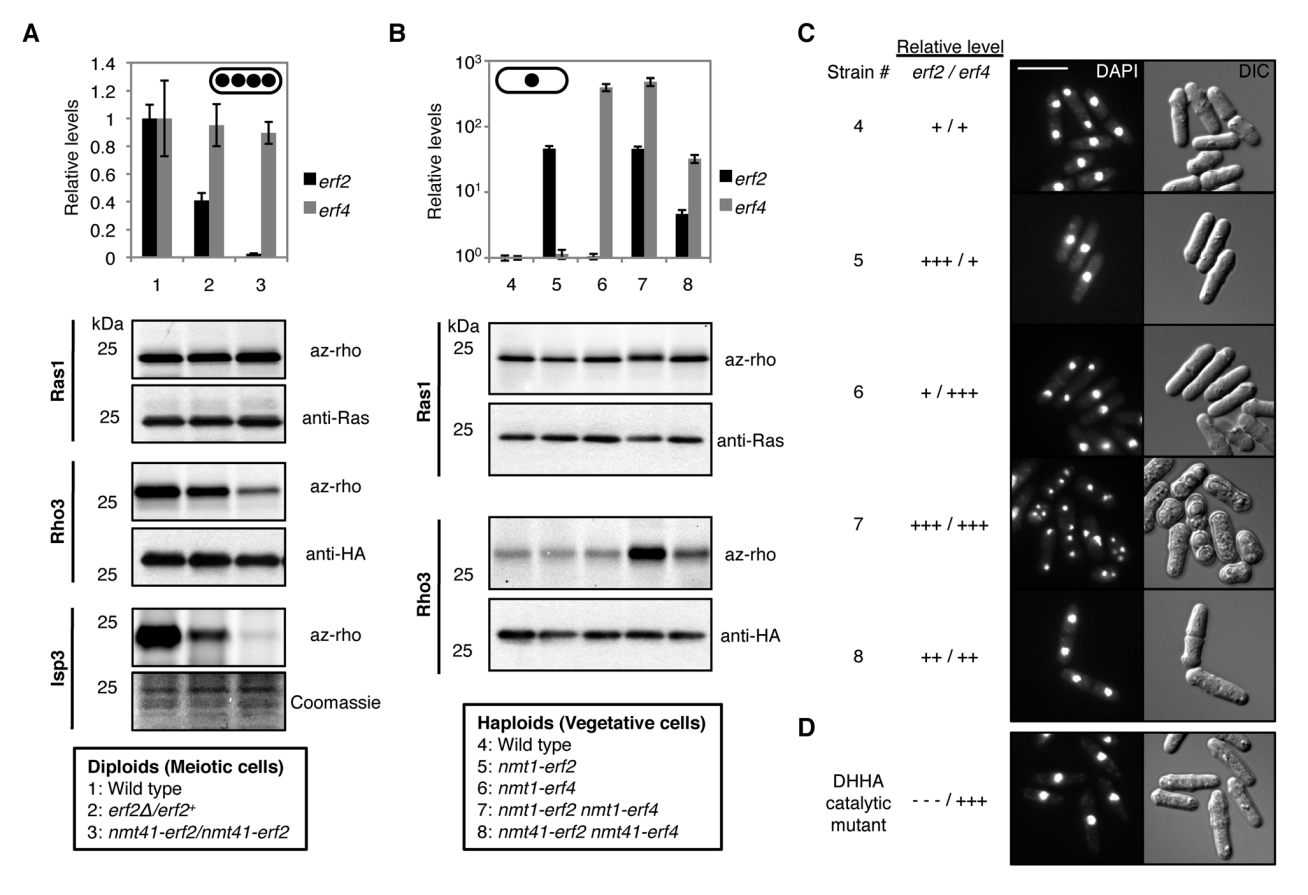
Quantitative control of protein palmitoylation by varying palmitoyltransferase levels affects meiotic entry. (A) Labels are indicated in box. qPCR analysis of *erf2* and *erf4* transcripts normalized to *act1* mRNA levels in indicated *pat1-114/pat1-114* strains 8 h into synchronous meiosis (top panel). Data represent mean values of experimental triplicates ± SD Palmitoylation of cognate Erf2 substrates were analyzed from the same lysate of each strain (bottom panels). Ras1 and Rho3-HA_3_ were immunopurified and their levels were determined by Ras1 and HA immunoblots, respectively. Isp3 palmitoylation was monitored at the lysate level since it accounts for most of the fluorescence at ∼23 kDa. (B) Labels are indicated in box. Overexpression of *erf2* and/or *erf4* from thiamine-repressible *nmt* promoters in the indicated vegetatively growing *pat1-114* cells was achieved by switching them into thiamine-free medium for 24 h. qPCR analysis (top panel) as well as palmitoylation of Ras1 and Rho3 (bottom panels) were performed as described in (A). Isp3 is not expressed in vegetative cells. Cells were maintained at permissive temperature throughout this experiment. (C, D) DAPI (left) and DIC (right) images of indicated cells 96 h after *erf2* and/or *erf4* overexpression. The DHHC→DHHA catalytic *erf2* mutant was co-overexpressed with *erf4* from *nmt1* promoters. Scale bars, 10 µm.

To test this hypothesis, we first reduced *erf2* expression in diploid cells undergoing synchronized meiosis, achieving intermediate and low expression levels relative to wild type (Figure 4A). Although all three substrates require Erf2 for palmitoylation, Ras1, Rho3, and Isp3 were differentially modified as a consequence of altering *erf2* levels (Figure 4A). Ras1 was efficiently palmitoylated at low *erf2* expression levels, and its palmitoylation was unaltered at higher levels. In contrast, reducing *erf2* expression decreased Rho3 and Isp3 palmitoylation with different sensitivities. The fact that palmitoylation of Rho3 and Isp3 are greatly attenuated at low *erf2* levels suggests that the strong upregulation of *erf2* expression during meiosis is needed to establish the distinctive features of the meiotic palmitoylome. In addition, these results demonstrate that modulation of palmitoyltransferase levels can differentially alter palmitoylation of individual substrates.

If physiological control of Erf2–Erf4 abundance accounts for the palmitoylome changes observed upon meiotic induction, we predicted that increasing Erf2–Erf4 levels in haploid vegetative cells would yield a palmitoylation profile similar to meiotic cells. To test this, we focused on Ras1 and Rho3 as model substrates since *isp3* expression is restricted to meiotic cells (Figure 3D). Ras1 palmitoylation was insensitive to increases in Erf2–Erf4 levels (Figure 4B), consistent with our previous results in meiotic cells (Figure 4A). In contrast, while overexpression of *erf2* and *erf4* individually had no impact on Rho3 modification, we observed a striking increase in Rho3 palmitoylation when the effective Erf2–Erf4 palmitoyltransferase concentration was increased by co-overexpression of both subunits of the palmitoyltransferase complex (Figure 4B). Intermediate *erf2–erf4* expression resulted in a modest increase in Rho3 palmitoylation, indicating a dose-dependent function of palmitoyltransferase activity (Figure 4B). Importantly, although *erf2* and *erf4* were expressed from heterologous promoters, their levels were in fact within physiological range, with the highest and lowest levels being comparable to those attained by meiotic and vegetative cells, respectively (Figure 1D). These results demonstrate the control of palmitoyltransferase levels as a mechanism by which cells can finely shape palmitoylomes, with an impact on cellular processes such as meiosis.

### High Erf2 Activity Induces Meiosis in Cells Through Ectopic Rho3 Palmitoylation

**Figure 5:**
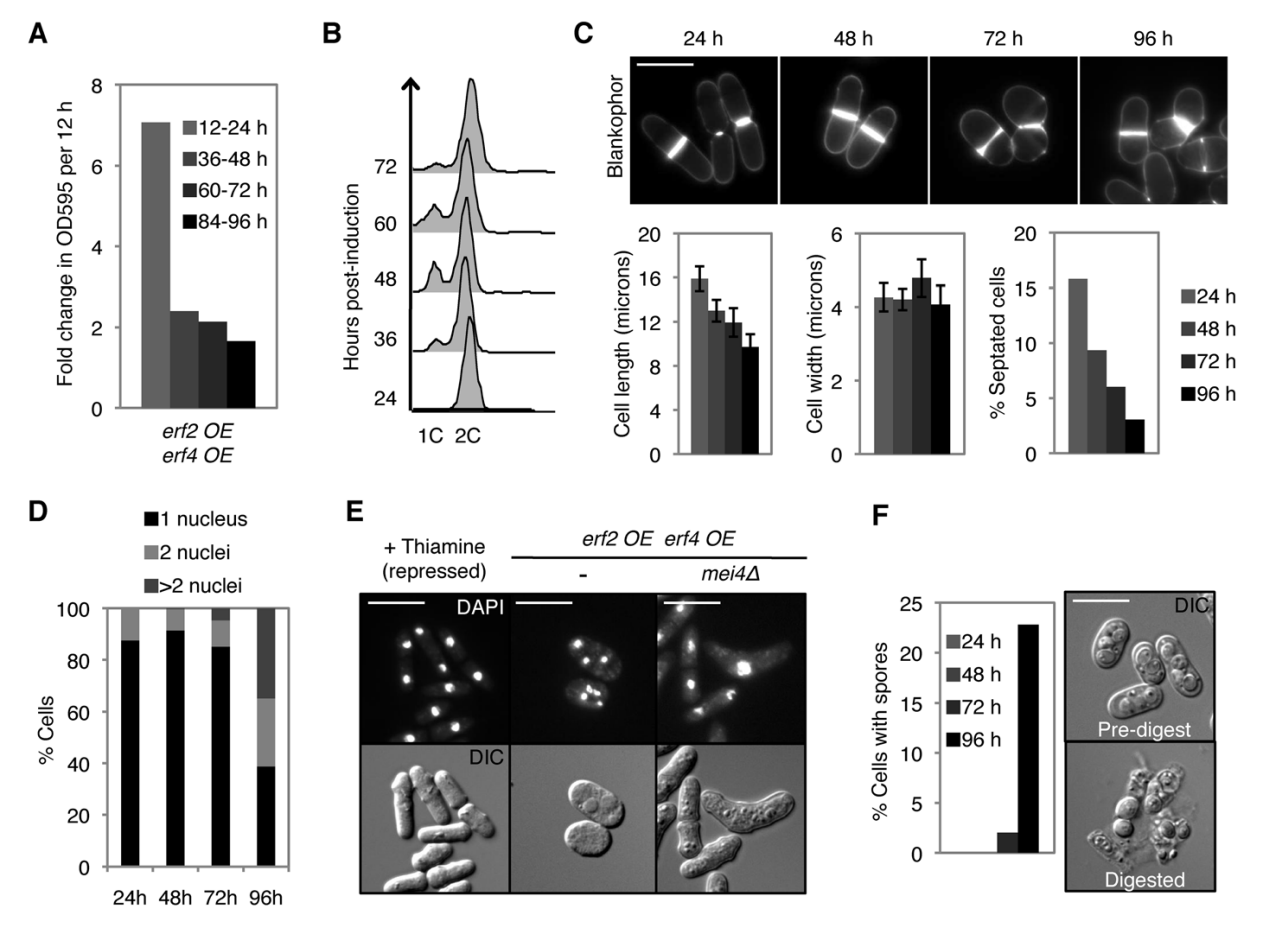
Overproduction of Erf2 and Erf4 in proliferating cells disrupts vegetative growth and induces aberrant meiosis and sporulation in***pat1-114*** **cells.** *erf2 OE erf4 OE* refers to strain 7 from Figure 4B that co-overexpresses *erf2* and *erf4* at high levels. These cells were grown in the presence of nutrients at permissive temperature, and co-overexpression of *erf2* and *erf4* was induced by switching cells to thiamine-free medium (see Materials and Methods). Indicated times or time intervals refer to time after induction of *erf2* and *erf4* co-overexpression. (A) Fold change in OD_595_ of cultures during indicated 12 h intervals. OD_595_ was maintained <0.6. (B) DNA content analysis. (C) Blankophor staining of cells (top panels). Dimensions of septated cells (cell length and width, *n* = 20) and percentage of septated cells (septation index, *n*≥200) were determined by measuring and counting blankophor-stained cells (bottom panels, left to right). Error bars, SD. (D) Percentage of cells with 1, 2, or >2 nuclei (*n*≥200) was determined by DAPI staining. (E) DAPI (top) and DIC (bottom) images of cells under conditions where *erf2* and *erf4* overexpression were repressed (+Thiamine) as well as 96 h postinduction of *erf2* and *erf4* co-overexpression. Scale bars, 10 µm. (F) Left panel, percentage of cells with spores (*n*≥200) at indicated times postinduction. Right panel, DIC images of cells 96 h postinduction before (top) and after (bottom) β-glucuronidase digestion, which specifically kills vegetative cells but not spores. Nonoverexpressing *pat1-114* cells continue vegetative growth under the same conditions (see Figure S5).

If palmitoyltransferase-level-mediated changes in the global protein palmitoylation are important for meiotic entry, then increasing Erf2–Erf4 levels in vegetative cells may be sufficient to promote this process. While no obvious phenotype was observed for *pat1^+^* cells (Figure S7), overexpression of *erf2* and *erf4* in proliferating haploid cells induced a striking meiotic phenotype under normal vegetative growth conditions (Figure 4C). In these cells, we observed a significant reduction in growth rate and proportion of dividing cells with time, accompanied by a reduction in cell length at division and a transient G1 delay (Figures 5A–C and S5A–C). In addition to being disrupted for vegetative growth, these *erf2* and *erf4* co-overexpressing cells subsequently underwent aberrant meiosis-like reductive cleavages, with a significant proportion of cells having >2 nuclei and containing spore-like structures (Figures 5D,E and S5D,E). These cells were indeed undergoing a meiotic program as deletion of *mei4*, which is required for meiotic but not mitotic divisions [46], suppressed the appearance of these multinucleated cells, resulting in misshapen cells arrested with a single nucleus (Figure 5E). This meiotic phenotype was dependent on high palmitoyltransferase levels, since cells maintained vegetative growth when *erf2* and *erf4* are expressed at low or intermediate levels (Figures 4C and 5E). Critically, this meiotic phenotype was not observed in cells co-overexpressing a catalytically inactive Erf2 mutant and Erf4 (Figure 4D) or in cells overproducing either Erf2 or Erf4 alone (Figure 4C), highlighting the requirement for a functional and active palmitoyltransferase complex. Together with the delay in meiotic entry observed in *erf2Δ* cells (Figure S6A,B), these results demonstrate that coordinated regulation of Erf2–Erf4 activity through changes in the expression of each subunit contributes to meiotic entry in fission yeast.

**Figure 6:**
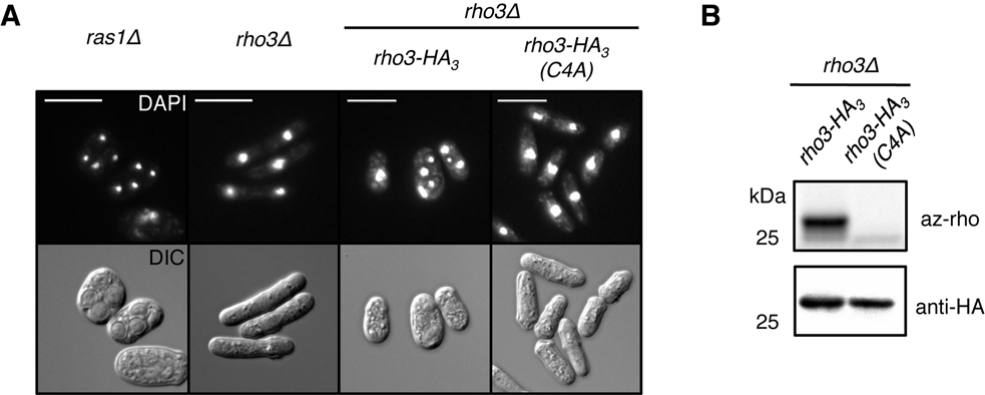
Palmitoylation-dependent Rho3 function is required for the Erf2-induced meiotic phenotype. (A) DAPI (top) and DIC (bottom) images of *erf2* and *erf4* co-overexpressing cells with indicated genotypes at 96 h postinduction. Vectors expressing internally tagged wild-type and mutant (Cys4→Ala) Rho3-HA_3_ proteins from the *nmt41* promoter were integrated into the chromosome of *rho3Δ* cells. Scale bars, 10 µm. (B) Alk-16-associated fluorescence of Rho3 and palmitoylation-deficient Rho3(C4A) (top panel). Western blots were probed for HA (bottom panel).

As the meiotic phenotype can be induced by Erf2–Erf4 overproduction, we reasoned that the Erf2–Erf4 substrates involved in triggering meiosis would have to be present in vegetative cells and sensitive to Erf2–Erf4 levels for their palmitoylation state. One such substrate is Rho3, whose palmitoylation state depends on *erf2* expression (Figure 4A,B). We found that Rho3, but not Ras1, is required for the meiotic phenotype triggered by Erf2–Erf4 overexpression in haploid cells (Figure 6A). This is clearly dependent on Rho3 palmitoylation, as cells expressing palmitoylation-deficient Rho3 failed to undergo Erf2-induced meiosis (Figure 6A,B). Genetic interaction between *erf2*, *erf4*, and *pat1*, which encodes the general inhibitor of meiosis, further supports the meiotic role of Erf2–Erf4 (Figure S7). Expression of *erf2* and *erf4* is upregulated in cells undergoing normal meiosis when encountering low nitrogen conditions [36], which normally initiates mating and subsequent meiosis, supporting our conclusion that high *erf2/erf4* levels promote meiotic entry. Overall, our results suggest that palmitoyltransferase-level-mediated modulation of the palmitoylome is a determinant of meiotic entry, likely via Rho3 function that is regulated by its palmitoylation state, closely interacting with the Pat1 kinase.

## Discussion

By analyzing multiple cognate substrates of a single palmitoyltransferase in a physiological context, our work offers a new perspective of how global protein palmitoylation can be regulated. We showed that modulation of Erf2–Erf4 activity levels underlies the differential modification of its cognate substrates in distinct cellular states, demonstrating the quantitative control of single or multiple palmitoyltransferase activities as a fundamental mechanism by which cells shape their palmitoylomes, with potentially important consequences on cellular function.

Our results uncover an intriguing possibility that a single palmitoyltransferase can regulate different cellular processes through distinct substrates depending on its levels. Indeed, high Erf2–Erf4 palmitoyltransferase levels promote or reinforce the meiotic state in fission yeast cells, consistent with the delay in meiotic entry observed for *erf2Δ* cells. The fact that *erf2* and *erf4* overexpression induces the meiotic phenotype in *pat1-114* cells where there is lower Pat1 kinase activity [47] but not in *pat1^+^* cells suggests that a sensitized background is needed to reveal the meiotic role of Erf2–Erf4 (Figure S7). This is not unexpected since in fission yeast, exit from vegetative growth and activation of the meiotic differentiation program are tightly regulated by a multilayered network of factors with many feedback loops, each contributing to stepwise inactivation of Pat1 and sequential derepression of meiosis [48]. It is unlikely that the induced meiotic phenotype is a result of nonspecific or general cellular stress since a variety of nutritional or environmental stresses alone failed to trigger meiosis in *pat1-114* cells (Figure S8). Moreover, despite the large number of genes that are shown to be regulated during meiosis or implicated in meiotic function [37],[49], only a few gene products have been shown to induce ectopic meiosis in vegetatively growing haploid wild type and/or *pat1-114* cells, and many have been demonstrated to be major meiotic determinants (Figure S9). Given that increasing a single palmitoyltransferase activity is sufficient to stimulate meiosis in *pat1-114* cells and that *erf2* and *erf4* expression is upregulated in wild-type cells undergoing normal meiosis in low nitrogen conditions [36], it is likely that Erf2–Erf4 modulates meiotic entry by contributing to Pat1 inactivation and derepression of meiosis. On the other hand, we cannot exclude the possibility that high Erf2–Erf4 levels reinforce the meiotic state once cells enter the meiotic program via positive feedback loops. It has been shown in budding yeast that commitment in meiosis does not occur until induction of middle genes [50].

Quantitative control of Rho3 palmitoylation by the Erf2–Erf4 palmitoyltransferase activity reveals a role for this family of small GTPases in meiosis. Rho3 in *S. pombe* regulates polarized cell growth and exocytosis [44],[45] and is involved in Golgi/endosome trafficking [51]. Delivery of cytoplasmic vesicles to discrete plasma-membrane domains is critical for establishing and maintaining cell polarity, neurite differentiation, and regulated exocytosis. Interestingly, Anastasia and co-workers showed in budding yeast that blocking membrane traffic causes a mitotic checkpoint, suggesting a link between mitotic entry and membrane growth [52]. If palmitoylation-dependent Rho3 function affects membrane trafficking, signals that are coupled to membrane growth may explain how Rho3 regulates meiotic entry in fission yeast. These signals may increase the concentration of receptors and effectors at the plasma membrane due to changes in membrane trafficking or cytoskeletal organization.

Our work has established the fission yeast *S. pombe*, with its relatively simple palmitoyltransferase network, as a robust and complementary model for understanding the basic control mechanisms and the function of protein palmitoylation. Palmitoylome analysis of various palmitoyltransferase-deficient mutants in budding yeast has provided clear evidence for enzyme-substrate specificity, with individual palmitoyltransferases showing a preference for substrates with common features; for example, ERF2–ERF4 substrates tend to be heterolipidated (e.g., prenylated or myristoylated) [20]. With Isp3 having an *N*-myristoylation motif (Figure 4D) and Ras1/Rho3 being prenylated, it appears that the orthologous Erf2–Erf4 palmitoyltransferase in fission yeast has the same substrate preference. Notably, the Erf2–Erf4:Ras1 palmitoyltransferase:substrate pairing in fission yeast is reminiscent of the ERF2–ERF4:RAS1/RAS2 and DHHC9-GCP16:H-/N-Ras pairs in budding yeast and human, respectively [16],[17],[53]. This suggests an evolutionary selection for specific cognate palmitoyltransferase:substrate pairs, which would be unlikely if palmitoyltransferases have extensive overlapping substrate preferences and were able to freely substitute for one another. The fact that ERF2–ERF4 also palmitoylates RHO3 in budding yeast [20] raises the possibility that in mammals, DHHC9-GCP16 may modify the large number of Rho GTPases with cysteines close to the N- or C-terminus that serve as potential palmitoylation sites [54]. If DHHC9-GCP16 indeed does palmitoylate Rho proteins, it would be interesting to determine if, like Erf2–Erf4, the palmitoyltransferase complex in mammals can discriminate between and differentially modify Ras and Rho proteins, potentially coordinating small GTPase function.

It remains to be determined how levels of a palmitoyltransferase affect its substrate specificity. One possibility is a difference in catalytic efficiency (*k*_cat_/*K*_M_), and this is supported by the discovery of specificity determinants on both the palmitoyltransferases and their substrates that dictate enzyme-substrate interactions [22],[55]. In addition, level-dependent changes in palmitoyltransferase localization might alter substrate availability. For example, DHHC2 translocates to postsynaptic membranes upon neuronal stimulation, where it increases palmitoylation and synaptic targeting of PSD-95 [6]. In our study, however, changes in palmitoyltransferase localization are unlikely to account for the difference in Ras1 and Rho3 palmitoylation since both substrates are localized to the same compartments in vegetative cells [38],[44]. Alternatively, it may be a result of titration by competing cellular factors that target substrates differentially [4],[14],[56]–[58].

As global proteomic studies rapidly expand the known list of proteins that are reversibly modified with this lipid moiety [13],[20],[35],[59],[60], palmitoylation is poised to be a major cellular regulator. This is supported by expression profiling experiments in flies and humans in which transcript levels of specific palmitoyltransferases vary widely across tissues [23],[24]. Neuronal differentiation signals were found to induce palmitoyltransferase degradation through the ubiquitin-proteasome pathway [61]. In addition, palmitoyltransferase overexpression is associated with a variety of human cancers and induces cellular transformation [29],[30],[62],[63]. To our knowledge, our study provides the first evidence that differential protein palmitoylation as a result of regulated palmitoyltransferase levels can influence cellular behavior. Although the palmitoylation machinery is more elaborate in multicellular eukaryotes, the fact that Ras and Rho GTPases are essential for cellular differentiation and development in mammals [54] as well as the conservation of palmitoyltransferase function and specific palmitoyltransferase:substrate pairs between yeast and humans [17],[20],[53] indicate that our conclusions provide fundamental insights for regulatory roles of palmitoylation in major developmental transitions in metazoa.

## Materials and Methods

### Strains and Growth Conditions

Standard media and methods were used [64],[65]. Strains used in this study are listed in Table S1. All experiments were carried out in minimal medium (EMM) and minimal medium plus supplements (EMM4S) for prototrophic and auxotrophic strains, respectively, at 25°C unless otherwise noted. When applicable, strains were generated by tetrad dissection and validated by marker segregation or PCR. Deletion strains, strains expressing *erf2-HA_3_*, *isp3-HA_3_*, and those expressing *erf2* and *erf4* from *nmt* promoters were constructed by PCR integration [66]. For Rho3, the HA_3_-tag sequence was inserted in-frame 42 bp upstream of its termination codon by PCR integration. Functional rescue in *erf2Δ* cells was performed using the pDUAL plasmid (RIKEN BRC, Japan) expressing *YFP-FLAG-His_6_-erf2* under the *nmt41* promoter, which was linearized and integrated into the *leu1* locus. The Quikchange XL II kit (Stratagene) was used for site-directed mutagenesis of the *erf2 DHHC* motif. Homozygous diploids were obtained by incubating midlog cultures of haploid cells with 20 µg/ml Carbendazim (Sigma) for 4.5 h at 25°C and screening colonies on YES+phloxin B plates.

### Synchronized Pat1-Driven Meiosis

Pat1-driven meiosis in homozygous diploid *h-/h- pat1-114/pat1-114* cells was carried out as described [37]. Midlog cultures grown in EMM, which contains 0.5% NH_4_Cl, were filter-washed three times with nitrogen-free minimal medium (EMM-N) using the Microfil filtration system (Millipore), and resuspended in EMM-N for 14 h at 25°C. Meiosis was induced by shifting the cultures to the restrictive temperature of 34°C in the presence of 0.05% NH_4_Cl. t = 0 is defined as the time of the temperature shift.

### *erf2* and/or *erf4* Overexpression From Thiamine-Repressible *nmt* Promoters

Cultures were grown to midlog in EMM plus 10 mg/ml thiamine at 25°C. All cells proliferate normally under these conditions. *erf2* and/or *erf4* expression from *nmt* promoters was induced by filter-washing the cells three times with EMM as described above. The cells were resuspended in EMM and growth was maintained at 25°C. The cultures were diluted with EMM approximately every 12 h for the duration of each experiment to keep OD_595_<0.6 and in the presence of nutrients. t = 0 h is defined as the time of thiamine removal.

### Flow Cytometry

DNA content was analyzed by flow cytometry using ethanol-fixed and propidium iodide-stained cells (2 µg/mL propidium iodide in 50 mM sodium citrate) on a BD FACS Calibur and analyzed using FlowJo software.

### Cell Size Measurements and DNA Staining

For cell size measurements, live cells were stained with Blankophore (MP Biomedicals). For DNA staining, ethanol-fixed cells were stained with DAPI. Images were acquired in Metamorph (MDS Analytical Technologies) using an Axioplan 2 microscope (Carl Zeiss) and a CoolSNAP HQ camera (Roper Scientific). Cell size measurements were obtained using the Pointpicker plug-in of Image J (National Institute of Health).

### Quantitative RT-PCR

Total RNA was extracted using acidic phenol, DNase I-treated, and purified with the RNeasy kit (Qiagen). RNA concentration was quantified and its integrity was determined by agarose gel electrophoresis. cDNA was synthesized using random hexamers and the SuperScriptIII First Strand Synthesis SuperMix (Invitrogen). Relative quantification of cDNA was carried out in triplicate for each independent experiment using qPCR MasterMix Plus for SYBR Green (Applied Biosystems) on an ABI 7900 Real-Time PCR system. Primers used for quantitative PCR are listed in Table S3. Standard curves were generated using at least six 2-fold serial dilutions of a control sample and values within the linear exponential phase were used to calculate relative concentrations after normalization to the endogenous actin controls.

### Metabolic Labeling and Preparation of Cell Lysates

Cells were labeled with 10 µM of alk-16 (20–50 mM DMSO stock) for 15 min, washed once with PBS prior to liquid nitrogen freezing and storage at −80°C. For inhibitor experiments, cells were preincubated for 30 min with either 200 µg/mL CHX or 200 µM 2BP, which were maintained in the cultures during alk-16 labeling. Competition experiments were carried out with various palmitate concentrations in the cultures during metabolic labeling. To prepare cell lysates, Brij lysis buffer (1% (v/v) Brij-97, 150 mM NaCl, 50 mM triethanolamine pH 7.4, 5× concentration of Roche EDTA-free protease inhibitor cocktail, 10 mM PMSF) and acid-washed glass beads (Sigma) were added to the frozen yeast cell pellets, which were lysed (3×20 s) using the Fastprep homogenizer (Thermo Scientific) at 4 min intervals to avoid overheating. Lysates were spun at 1,000 g for 5 min to remove cellular debris. Typical lysate protein concentrations of 5–10 mg/mL were obtained, as quantified using the BCA assay (Pierce).

### Immunoprecipitations and Bioorthogonal Labeling

For analyses of whole cell lysates, 50 µg of protein was diluted with Brij lysis buffer to a final volume of 44.5 µL, to which 5.5 µL of freshly mixed Cu^I^-catalyzed azide-alkyne cycloaddition (CuAAC) reagents were added. The CuAAC reagents consisted of 1 µL az-Rho (5 mM stock solution in DMSO), 1 µL tris(2-carboxyethyl)phosphine hydrochloride (TCEP) (50 mM freshly prepared stock solution in deionized water), 2.5 µL tris[(1-benzyl-1*H*-1,2,3-triazol-4-yl)methyl]amine (TBTA) (2 mM stock solution in 1∶4 DMSO∶t-butanol), and 1 µL CuSO_4_·5H_2_O (50 mM freshly prepared stock solution in deionized water). After 1 h at room temperature, proteins were methanol-chloroform precipitated to remove excess CuAAC reagents. The protein pellets were air-dried and resuspended in SDS buffer (4% (w/v) SDS, 150 mM NaCl, 50 mM triethanolamine pH 7.4) by sonication before SDS-PAGE. For Ras1 and HA immunoprecipitations, 1–3 mg of cell lysate was added to 4 µg anti-Ras antibody (Ras10, Millipore) with 25 µL Protein A agarose (Roche) or to 15 µL of anti-HA antibody–conjugated agarose (3F10, Roche), respectively. After 2 h incubation with rocking at 4°C, the beads were washed three times with ice-cold RIPA buffer (1% (v/v) Triton X-100, 1% (w/v) sodium deoxycholate, 0.1% (w/v) SDS, 50 mM triethanolamine pH 7.4, 150 mM NaCl). The washed beads were resuspended in 20 µL of PBS and 2.25 µL freshly mixed CuAAC reagents described above. The beads were then washed three times with ice-cold RIPA buffer prior to boiling in SDS buffer for SDS-PAGE.

### In-Gel Fluorescence Scanning and Western Blots

Fluorescence gels were visualized on a Typhoon 9400 variable mode imager (GE Healthcare) at excitation 532 nm/emission 580 nm. For Western blotting, proteins separated by SDS-PAGE were transferred to nitrocellulose membranes and probed with the following antibodies: anti-Ras (Ras10, Millipore), anti-tubulin (Tat1, gift from Keith Gull), and anti-HA (3F10, Roche). To avoid visualizing the light chain band in anti-Ras1 immunoblots of Ras1 immunopurifications, an Fc-specific anti-mouse-HRP secondary antibody (A2544, Sigma) was used. Blots were developed using the enhanced chemiluminescence kit (GE Healthcare). Images were processed using Image J.

### Affinity Enrichment and Mass Spectrometry

CuAAC reagents (az-azo-biotin) [41] were added to 10 mg cell lysate at the same concentrations described above. Proteins were methanol-precipitated and resulting air-dried protein pellets were resuspended in 1 mL SDS buffer containing 10 mM EDTA by sonication. The mixture was diluted 1∶3 with Brij lysis buffer and incubated with 100 µL of washed streptavidin agarose resin (Pierce) for 1 h on a nutating mixer at room temperature. The beads were then washed once with 0.2% (w/v) SDS in PBS, three times with PBS, and twice with 250 mM ammonium bicarbonate (ABC). Beads were resuspended in 500 µL 8 M urea, and reactive cysteines were alkylated by addition of 25 µL 200 mM TCEP and 25 µL 400 mM iodoacetamide for 30 min. The beads were washed twice with 50 mM ABC. Two sequential elutions of proteins from the resin were performed by incubating the beads with 250 µL of 25 mM sodium dithionite in 50 mM ABC with 0.1% (w/v) SDS for 30 min each. Proteins were concentrated using YM-10 Centricons (Millipore), dried in a speed vac, and separated by SDS-PAGE. Upon staining with Coomassie blue, each lane was cut into 10 slices for trypsin digestion and peptide extraction. Extracted peptides were dried and resuspended in 0.1% (v/v) trifluoroacetic acid for mass spectrometry identification. Acquired MS/MS spectra were analyzed using the Sequest search engine to identify proteins from the primary sequence database obtained from the *S. pombe* GeneDB. Exported Sequest results were analyzed using Scaffold (Proteome Software).

## Supporting Information

Figure S1 ***S. pombe*** **meiosis is a specialized and tightly regulated process.** (A) Fission yeast cells normally proliferate as haploids through the mitotic cell cycle (right cycle, shaded), replicating their genome (S phase) and dividing the genetic material equally between two daughter cells during mitosis (M phase). When nutrients such as nitrogen become limiting, cells may enter an alternate meiotic differentiation pathway that is distinct from the mitotic cycle (left cycle). Haploid cells transiently arrest in G1 and conjugate with cells of opposite mating types to form a diploid zygote. These diploids replicate their genome (meiotic S phase) and then undergo two successive nuclear divisions (Meiosis I and II) to yield four haploid nuclei that mature into spores, completing meiosis. Each of these haploid spores germinates into normally dividing cells when favorable conditions return. (B) In *S. pombe*, the Mei2 master regulator governs the switch from the mitotic to meiotic cycle. Mei2 integrates extracellular cues (stress, nutrients, and pheromones) primarily through Ste11, and drives meiosis. Mei2 function is tightly regulated by the Pat1 kinase, which inactivates both Mei2 and Ste11, thereby preventing meiotic entry during the mitotic cell cycle [67]. Together, Pat1 and Mei2 constitute the core mitosis-meiosis switch in fission yeast. Working in concert, these key cellular factors integrate environmental cues and control entry into meiosis. The temperature-sensitive *pat1-114* allele of Pat1 allows the induction of synchronous meiosis regardless of normal biological cues [32],[33]. Haploid and diploid cells harboring this mutation can be induced to undergo meiosis in a timely and predictable manner that facilitates characterization of the process by shifting the cultures from permissive to restrictive temperatures. At permissive temperature, Pat1 is active in homozygous diploids. As such, Mei2 is inactivated and cells do not enter meiosis. Although *pat1-114* cells are more sensitive to pheromones [47], they proliferate normally and require the normal biological cues to enter meiosis at permissive temperature. On the other hand, at restrictive temperature, Pat1 is inactivated and derepression of Mei2 function induces spontaneous meiosis in *pat1-114* cells.
(TIF)

Figure S2 **Specific and sensitive bioorthogonal detection of protein palmitoylation and palmitoyltransferase activity** ***in vivo*** **in fission yeast.** (A) Schematic representation of the bioorthogonal detection protocol. Alk-16, alkyne-functionalized palmitate reporter; Az-rho, azide-functionalized rhodamine fluorophore; CuAAC, copper-catalyzed azide-alkyne cycloaddition. (B) In-gel fluorescent detection of alk-16-labeled proteins in lysates and immunopurified Ras1 (Ras1 IP). Protein load was determined by Coomassie blue staining of the gel. (C, D) Fluorescent detection of immunopurified Ras1, a known palmitoylated protein, from alk-16-labeled cells (top panels). Western blots were probed for Ras1 (bottom panels). Palmitate competed with alk-16 labeling in a dose-dependent manner (C). Ras1-associated fluorescence was greatly diminished by pre-incubating cells with a general palmitoylation inhibitor 2-bromopalmitate (2BP) or by selective post-CuAAC cleavage of palmitoylation-specific thioester linkages with hydroxylamine (NH_2_OH). Pretreatment of cells with protein synthesis inhibitor cycloheximide (CHX) had little effect on posttranslational Ras1 labeling by alk-16 (D).
(TIF)

Figure S3 **Erf2 and Erf4 expression is selectively regulated during sexual differentiation in fission yeast.** (A) Partial amino acid sequence alignments and consensus sequence of *S. pombe* palmitoyltransferases and phenotypes of null mutants [68]. (B) qPCR analysis of palmitoyltransferase transcripts in vegetative and meiotic *pat1-114/pat1-114* cells. *y*-axis, fold change, meiotic/vegetative levels. All transcript levels were normalized to *act1* mRNA. Error bars, SD.
(TIF)

Figure S4 **Enrichment and identification of Erf2 substrates that are selectively palmitoylated during meiosis.** (A) Schematic representation of the selective enrichment protocol of alk-16-modified proteins from cell lysates. Az-azo-biotin, Azide-functionalized biotin probe with an azobenzene cleavable linker; CuAAC, copper-catalyzed azide-alkyne cycloaddition. (B) Selective enrichment of Ras1 in Na_2_S_2_O_4_ elutions from lysates of cells metabolically labeled with alk-16 (bottom panel) over input lysates (top panel). Western blots were probed for Ras1. (C) Coomassie blue stain of proteins before (input, top panel) and after affinity enrichment/elution (bottom panel). Slices of the bottom gel were processed for gel-based mass spectrometry. (D) Amino acid sequences of Rho3 and Isp3, both of which were validated to be Erf2 substrates that are selectively palmitoylated in meiotic cells in Figure 3. Yellow, identified peptides; Green, modified amino acids in identified peptides (e.g., oxidation, carbamidomethylation).
(TIF)

Figure S5 **Ectopic meiosis in haploid** ***pat1-114*** **cells co-overexpressing** ***erf2*** **and** ***erf4*** **.** ***erf2 OE erf4 OE:*** **strain 7 from** **Figure 4C** **that co-overexpresses** ***erf2*** **and** ***erf4*** **at high levels.** These cells were grown at permissive temperature, and co-overexpression of *erf2* and *erf4* was induced by switching cells to thiamine-free medium (see Materials and Methods). Nonoverexpressing *pat1-114* cells (*erf2 erf4*) continue vegetative growth under these conditions. Indicated times or time intervals refer to time after the switch to thiamine-free medium. (A) Fold change in OD_595_ of cultures during indicated 12 h intervals. OD_595_ was maintained <0.6. (B) DNA content analysis. (C) Blankophor staining of cells (left panels). Scale bars, 10 µm. Dimensions of septated cells (cell length and width, *n* = 20, middle panels) and percentage of septated cells (septation index, *n*≥200, right panel) were determined by measuring and counting blankophor-stained cells. Error bars, SD. (D) Percentage of cells with 1, 2, or >2 nuclei (*n*≥200) was determined by DAPI staining of the indicated strains. (E) Percentage of cells with spores at indicated times postinduction (*n*≥200).
(TIF)

Figure S6 ***erf2Δ*** **cells are delayed in meiotic entry.** (A) Schematic representation of fission yeast sexual differentiation. Haploid cells conjugate to form a diploid zygote, which undergoes meiosis to yield four haploid nuclei that matures into spores. Pat1 kinase is a repressor of meiosis. For more details on *S. pombe* meiosis, see Figure S1. (B) DNA content analysis of indicated strains after meiotic induction by thermal inactivation of Pat1. (C) Percentage of cells with 1, 2, or >2 nuclei were determined by counting ≥200 DAPI-stained cells of the indicated strains at hourly intervals after meiotic induction (left panel). Representative DIC (middle panel) and DAPI (right panel) images of cells at indicated times. Scale bars, 10 µm. Synchronous meiosis in the indicated diploid *pat1-114/pat1-114* cells was induced by shifting nitrogen-starved cultures to a restrictive temperature (see Materials and Methods). Indicated times refer to the elapsed time after temperature shift.
(TIF)

Figure S7 **Erf2-Erf4 function in meiotic control is revealed in** ***pat1-114*** **cells.** DAPI and DIC staining of haploid cells at 96 h after induction of *erf2* and *erf4* co-overexpression. Cells were grown in the presence of nutrients at permissive temperature. Scale bars, 10 µm. Erf2–Erf4-induced meiosis is observed in *pat1-114* (top panels) but not *pat1^+^* cells (bottom panels). Our data suggest that Erf2–Erf4 function in meiotic control is unmasked in *pat1-114* cells where there is lower Pat1 kinase activity [47]. It is likely that high Erf2–Erf4 activity induces ectopic meiosis by either activating the Ste11-Mei2 pathway or inactivating Pat1.
(TIF)

Figure S8 ***pat1-114*** **cells do not enter meiosis when exposed to nutritional and other stresses.** DAPI and DIC images of *pat1-114* cells upon exposure to indicated stresses. Scale bars, 10 µm. (A) Nutritional stress. Cells were grown at high densities for 7 d at 25°C in minimal medium containing the indicated sole nitrogen source (NH_4_Cl, glutamate, proline) or in nitrogen-free minimal medium (N-free). Proline is considered to be a poor nitrogen source. (B) Osmotic stress. Cells were grown in minimal medium containing 1 M sorbitol, 0.4 M NaCl, or 1 M KCl for 5 d at 25°C. No meiotic cells were observed in these experiments.
(TIF)

Figure S9 **Gene products that induce ectopic meiosis in haploid** ***S. pombe*** **cells.** (A) Of the thousands of genes that significantly regulated during meiosis [37],[49], the listed gene products are demonstrated to induce meiosis in haploid cells, highlighting their regulatory roles in the important cellular transition. The exact molecular mechanisms by which some of these gene products modulate meiotic entry remain to be elucidated. Unless otherwise stated, cells are *pat1^+^*. (B) Signaling network that regulates fission yeast meiotic entry. In *S. pombe*, the Mei2 master regulator governs the switch from the mitotic to meiotic cycle. Mei2 integrates extracellular cues (stress, nutrients, and pheromones) primarily through Ste11, and drives meiosis. Mei2 function is tightly regulated by the Pat1 kinase, which inactivates both Mei2 and Ste11, thereby preventing meiotic entry during the mitotic cell cycle [67]. Together, Pat1 and Mei2 constitute the core mitosis-meiosis switch in fission yeast. Under physiological conditions, complete inactivation of Pat1 and meiotic induction strictly requires the stoichiometric inhibitor Mei3, which is only expressed in heterozygous diploids formed after successful conjugation [69]–[71]. Working in concert, these key cellular factors integrate environmental cues and control cellular entry into meiosis. For simplification, many factors and interactions are omitted in this representation. Meiotic activators and repressors are in green and red, respectively. Dashed lines represent proposed mechanism of Erf2–Erf4 in this study. Erf2 and Erf4 expression is significantly upregulated in meiotic cells compared to vegetative cells, and elevated Erf2–Erf4 levels promote the meiotic state by activating Mei2 and/or inactivating Pat1. Dotted lines represent either direct or indirect interactions.
(TIF)

Table S1 ***S. pombe*** **strains used in this study.**
(DOC)

Table S2 **Candidate Erf2 substrates that are selectively palmitoylated during meiosis.** Proteins were sorted in by their net spectral counts over the DMSO samples in meiotic homozygous diploid *erf2^+^/erf2^+^ pat1-114/pat1-114* cells. Based on molecular weight and enrichment over the DMSO control, Isp3 and Rho3 were chosen and biochemically validated in this study.
(DOC)

Table S3 **Oligonucleotide primers used in quantitative RT-PCR.**
(DOC)

## Abbreviations

DHHC: Asp-His-His-Cys motif
