# Supplementary figures and images for: Quantitative Control of Protein S-Palmitoylation Regulates Meiotic Entry in Fission Yeast
Source: PLoS Biol. 2013 Jul 2;11(7):e1001597. doi: 10.1371/journal.pbio.1001597 (PMC3699447; doi:10.1371/journal.pbio.1001597)

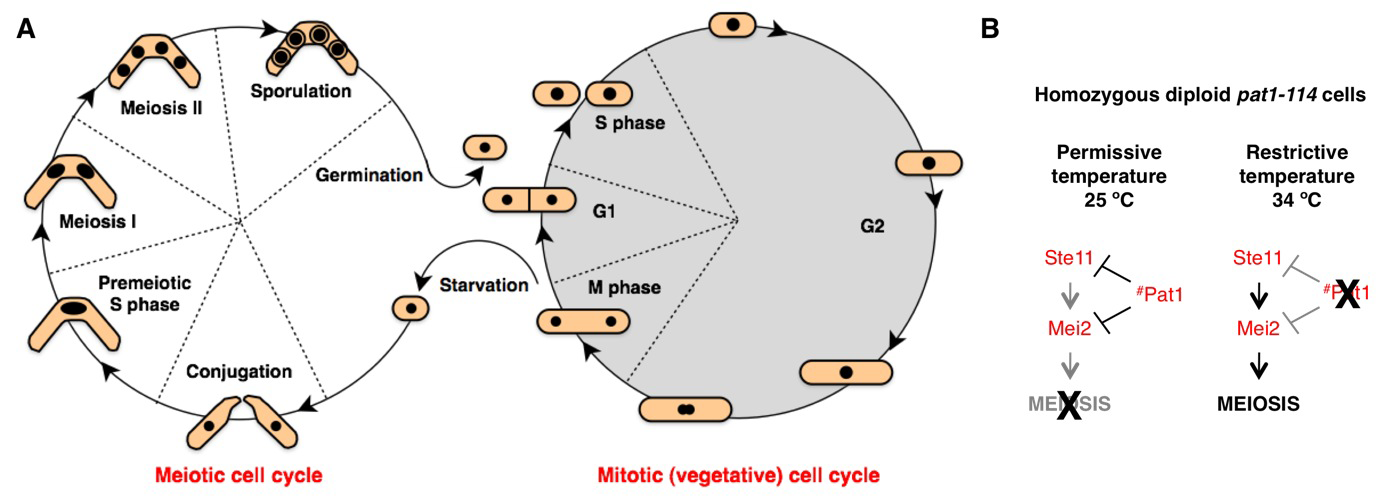

Supplement: Figure S1 — S. pombe meiosis is a specialized and tightly regulated process. (A) Fission yeast cells normally proliferate as haploids through the mitotic cell cycle (right cycle, shaded), replicating their genome (S phase) and dividing the genetic material equally between two daughter cells during mitosis (M phase). When nutrients such as nitrogen become limiting, cells may enter an alternate meiotic differentiation pathway that is distinct from the mitotic cycle (left cycle). Haploid cells transiently arrest in G1 and conjugate with cells of opposite mating types to form a diploid zygote. These diploids replicate their genome (meiotic S phase) and then undergo two successive nuclear divisions (Meiosis I and II) to yield four haploid nuclei that mature into spores, completing meiosis. Each of these haploid spores germinates into normally dividing cells when favorable conditions return. (B) In S. pombe, the Mei2 master regulator governs the switch from the mitotic to meiotic cycle. Mei2 integrates extracellular cues (stress, nutrients, and pheromones) primarily through Ste11, and drives meiosis. Mei2 function is tightly regulated by the Pat1 kinase, which inactivates both Mei2 and Ste11, thereby preventing meiotic entry during the mitotic cell cycle [67]. Together, Pat1 and Mei2 constitute the core mitosis-meiosis switch in fission yeast. Working in concert, these key cellular factors integrate environmental cues and control entry into meiosis. The temperature-sensitive pat1-114 allele of Pat1 allows the induction of synchronous meiosis regardless of normal biological cues [32],[33]. Haploid and diploid cells harboring this mutation can be induced to undergo meiosis in a timely and predictable manner that facilitates characterization of the process by shifting the cultures from permissive to restrictive temperatures. At permissive temperature, Pat1 is active in homozygous diploids. As such, Mei2 is inactivated and cells do not enter meiosis. Although pat1-114 cells are more sens [file pbio.1001597.s001.tif]

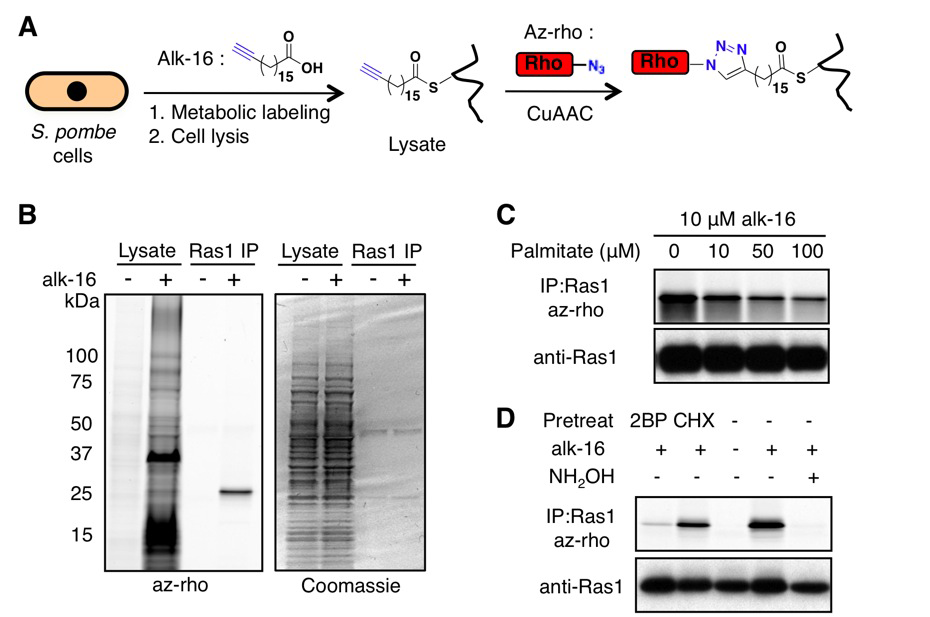

Supplement: Figure S2 — Specific and sensitive bioorthogonal detection of protein palmitoylation and palmitoyltransferase activity in vivo in fission yeast. (A) Schematic representation of the bioorthogonal detection protocol. Alk-16, alkyne-functionalized palmitate reporter; Az-rho, azide-functionalized rhodamine fluorophore; CuAAC, copper-catalyzed azide-alkyne cycloaddition. (B) In-gel fluorescent detection of alk-16-labeled proteins in lysates and immunopurified Ras1 (Ras1 IP). Protein load was determined by Coomassie blue staining of the gel. (C, D) Fluorescent detection of immunopurified Ras1, a known palmitoylated protein, from alk-16-labeled cells (top panels). Western blots were probed for Ras1 (bottom panels). Palmitate competed with alk-16 labeling in a dose-dependent manner (C). Ras1-associated fluorescence was greatly diminished by pre-incubating cells with a general palmitoylation inhibitor 2-bromopalmitate (2BP) or by selective post-CuAAC cleavage of palmitoylation-specific thioester linkages with hydroxylamine (NH2OH). Pretreatment of cells with protein synthesis inhibitor cycloheximide (CHX) had little effect on posttranslational Ras1 labeling by alk-16 (D). (TIF) [file pbio.1001597.s002.tif]

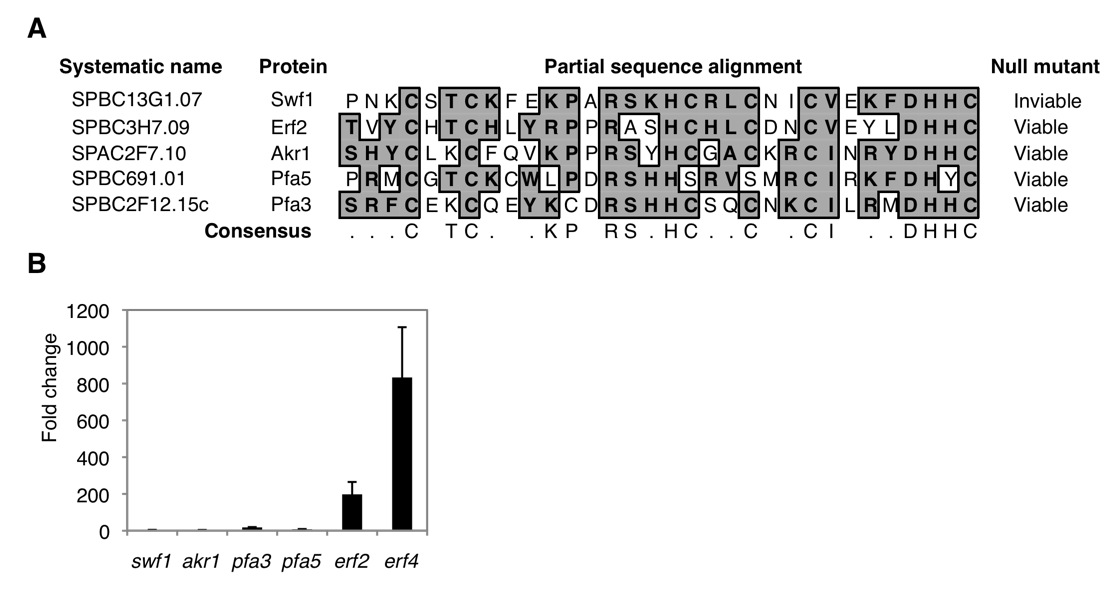

Supplement: Figure S3 — Erf2 and Erf4 expression is selectively regulated during sexual differentiation in fission yeast. (A) Partial amino acid sequence alignments and consensus sequence of S. pombe palmitoyltransferases and phenotypes of null mutants [68]. (B) qPCR analysis of palmitoyltransferase transcripts in vegetative and meiotic pat1-114/pat1-114 cells. y-axis, fold change, meiotic/vegetative levels. All transcript levels were normalized to act1 mRNA. Error bars, SD. (TIF) [file pbio.1001597.s003.tif]

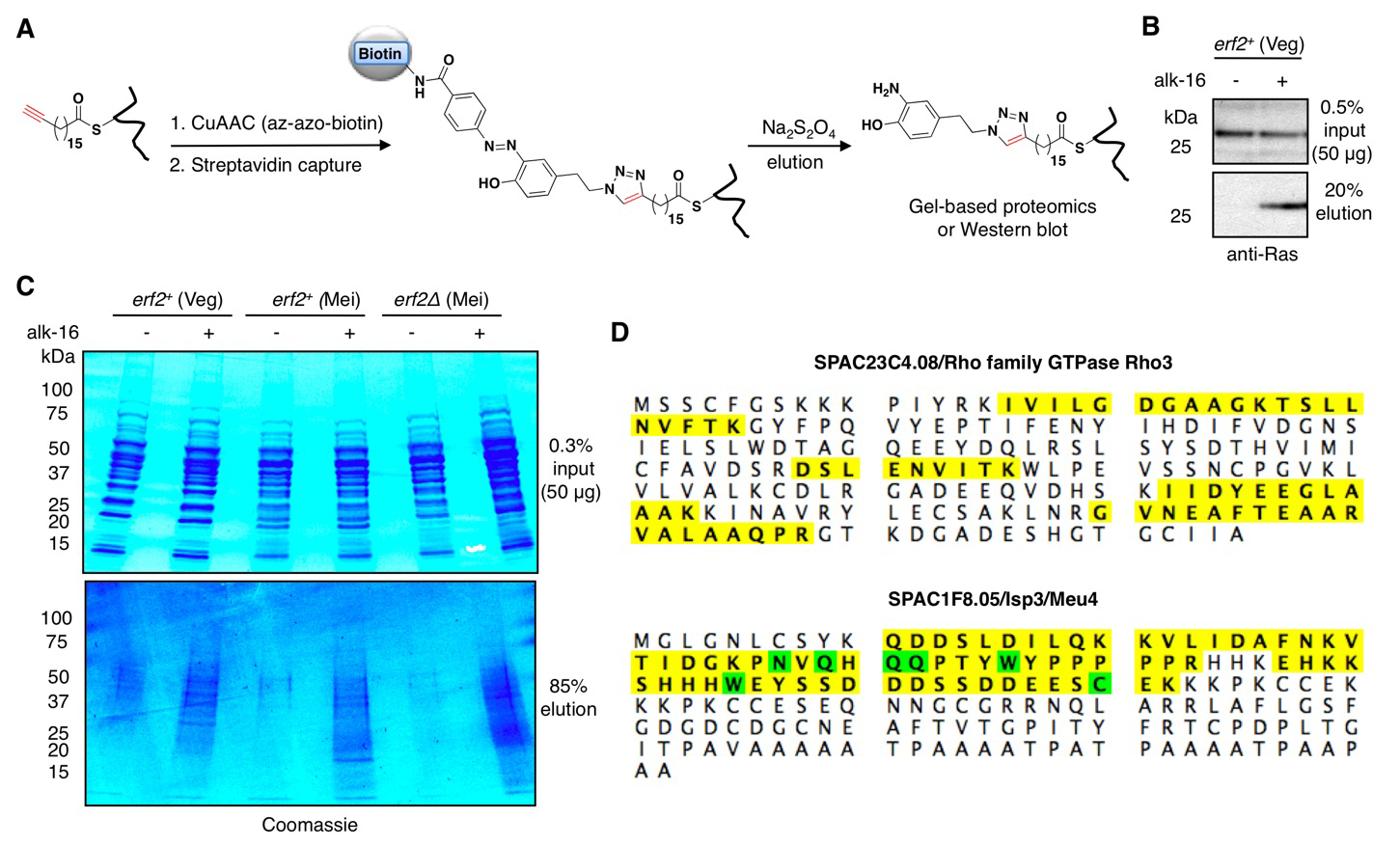

Supplement: Figure S4 — Enrichment and identification of Erf2 substrates that are selectively palmitoylated during meiosis. (A) Schematic representation of the selective enrichment protocol of alk-16-modified proteins from cell lysates. Az-azo-biotin, Azide-functionalized biotin probe with an azobenzene cleavable linker; CuAAC, copper-catalyzed azide-alkyne cycloaddition. (B) Selective enrichment of Ras1 in Na2S2O4 elutions from lysates of cells metabolically labeled with alk-16 (bottom panel) over input lysates (top panel). Western blots were probed for Ras1. (C) Coomassie blue stain of proteins before (input, top panel) and after affinity enrichment/elution (bottom panel). Slices of the bottom gel were processed for gel-based mass spectrometry. (D) Amino acid sequences of Rho3 and Isp3, both of which were validated to be Erf2 substrates that are selectively palmitoylated in meiotic cells in Figure 3. Yellow, identified peptides; Green, modified amino acids in identified peptides (e.g., oxidation, carbamidomethylation). (TIF) [file pbio.1001597.s004.tif]

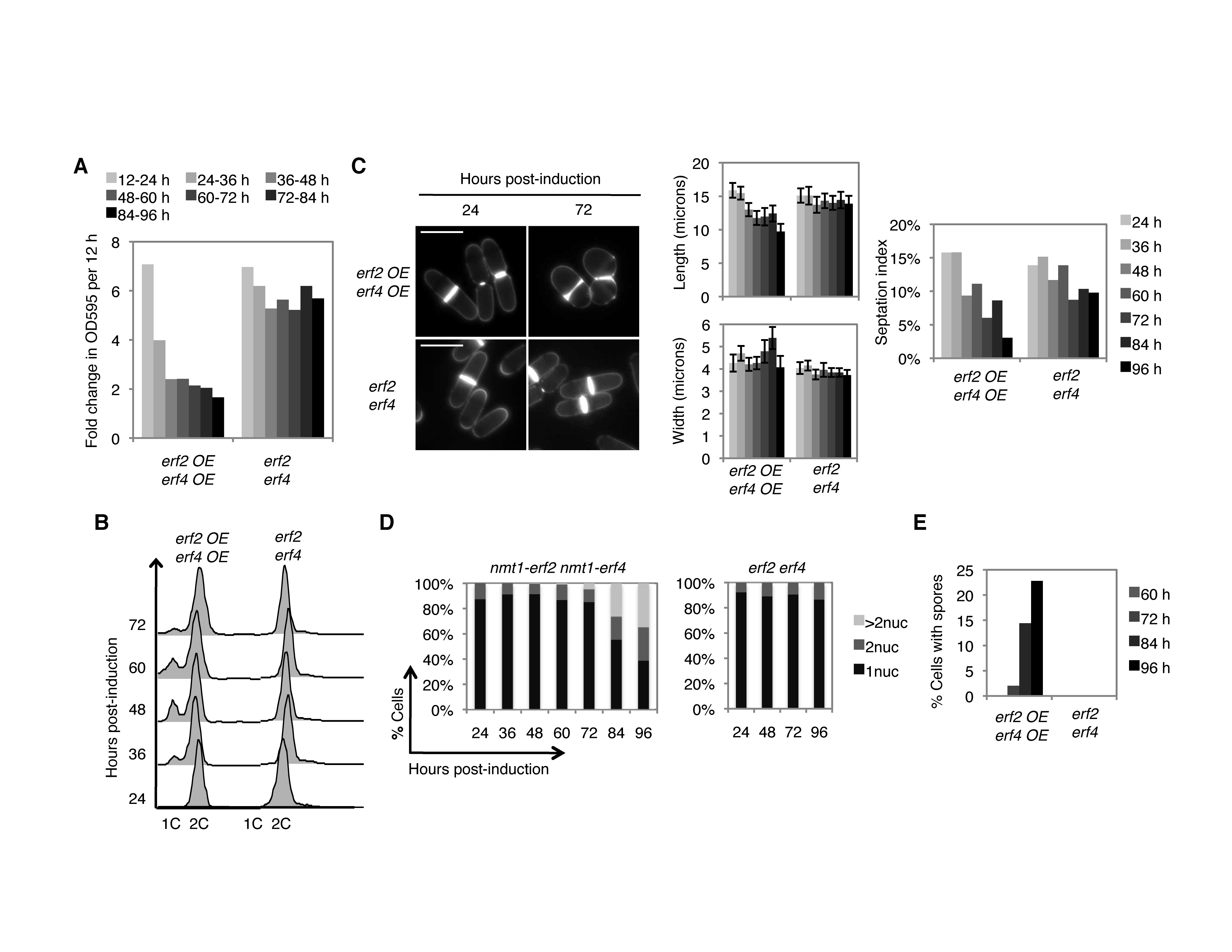

Supplement: Figure S5 — Ectopic meiosis in haploid pat1-114 cells co-overexpressing erf2 and erf4 . erf2 OE erf4 OE: strain 7 from Figure 4C that co-overexpresses erf2 and erf4 at high levels. These cells were grown at permissive temperature, and co-overexpression of erf2 and erf4 was induced by switching cells to thiamine-free medium (see Materials and Methods). Nonoverexpressing pat1-114 cells (erf2 erf4) continue vegetative growth under these conditions. Indicated times or time intervals refer to time after the switch to thiamine-free medium. (A) Fold change in OD595 of cultures during indicated 12 h intervals. OD595 was maintained <0.6. (B) DNA content analysis. (C) Blankophor staining of cells (left panels). Scale bars, 10 µm. Dimensions of septated cells (cell length and width, n = 20, middle panels) and percentage of septated cells (septation index, n≥200, right panel) were determined by measuring and counting blankophor-stained cells. Error bars, SD. (D) Percentage of cells with 1, 2, or >2 nuclei (n≥200) was determined by DAPI staining of the indicated strains. (E) Percentage of cells with spores at indicated times postinduction (n≥200). (TIF) [file pbio.1001597.s005.tif]

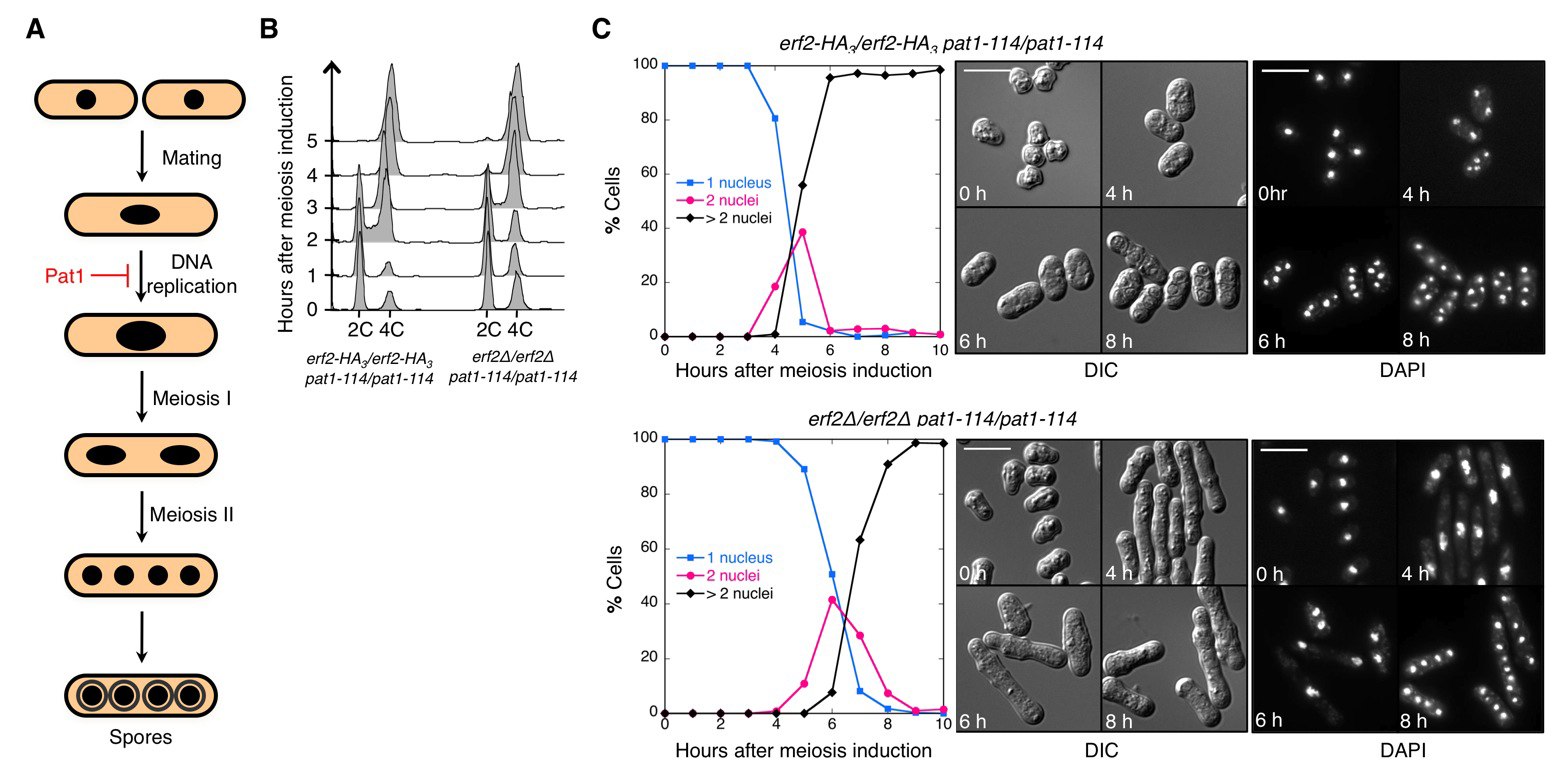

Supplement: Figure S6 — erf2Δ cells are delayed in meiotic entry. (A) Schematic representation of fission yeast sexual differentiation. Haploid cells conjugate to form a diploid zygote, which undergoes meiosis to yield four haploid nuclei that matures into spores. Pat1 kinase is a repressor of meiosis. For more details on S. pombe meiosis, see Figure S1. (B) DNA content analysis of indicated strains after meiotic induction by thermal inactivation of Pat1. (C) Percentage of cells with 1, 2, or >2 nuclei were determined by counting ≥200 DAPI-stained cells of the indicated strains at hourly intervals after meiotic induction (left panel). Representative DIC (middle panel) and DAPI (right panel) images of cells at indicated times. Scale bars, 10 µm. Synchronous meiosis in the indicated diploid pat1-114/pat1-114 cells was induced by shifting nitrogen-starved cultures to a restrictive temperature (see Materials and Methods). Indicated times refer to the elapsed time after temperature shift. (TIF) [file pbio.1001597.s006.tif]

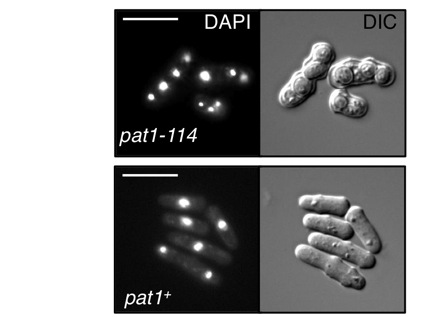

Supplement: Figure S7 — Erf2-Erf4 function in meiotic control is revealed in pat1-114 cells. DAPI and DIC staining of haploid cells at 96 h after induction of erf2 and erf4 co-overexpression. Cells were grown in the presence of nutrients at permissive temperature. Scale bars, 10 µm. Erf2–Erf4-induced meiosis is observed in pat1-114 (top panels) but not pat1+ cells (bottom panels). Our data suggest that Erf2–Erf4 function in meiotic control is unmasked in pat1-114 cells where there is lower Pat1 kinase activity [47]. It is likely that high Erf2–Erf4 activity induces ectopic meiosis by either activating the Ste11-Mei2 pathway or inactivating Pat1. (TIF) [file pbio.1001597.s007.tif]

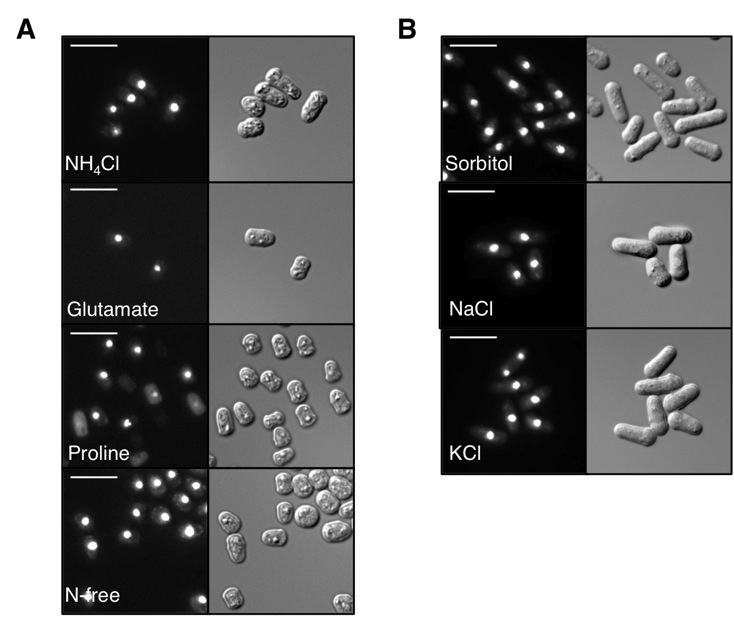

Supplement: Figure S8 — pat1-114 cells do not enter meiosis when exposed to nutritional and other stresses. DAPI and DIC images of pat1-114 cells upon exposure to indicated stresses. Scale bars, 10 µm. (A) Nutritional stress. Cells were grown at high densities for 7 d at 25°C in minimal medium containing the indicated sole nitrogen source (NH4Cl, glutamate, proline) or in nitrogen-free minimal medium (N-free). Proline is considered to be a poor nitrogen source. (B) Osmotic stress. Cells were grown in minimal medium containing 1 M sorbitol, 0.4 M NaCl, or 1 M KCl for 5 d at 25°C. No meiotic cells were observed in these experiments. (TIF) [file pbio.1001597.s008.tif]

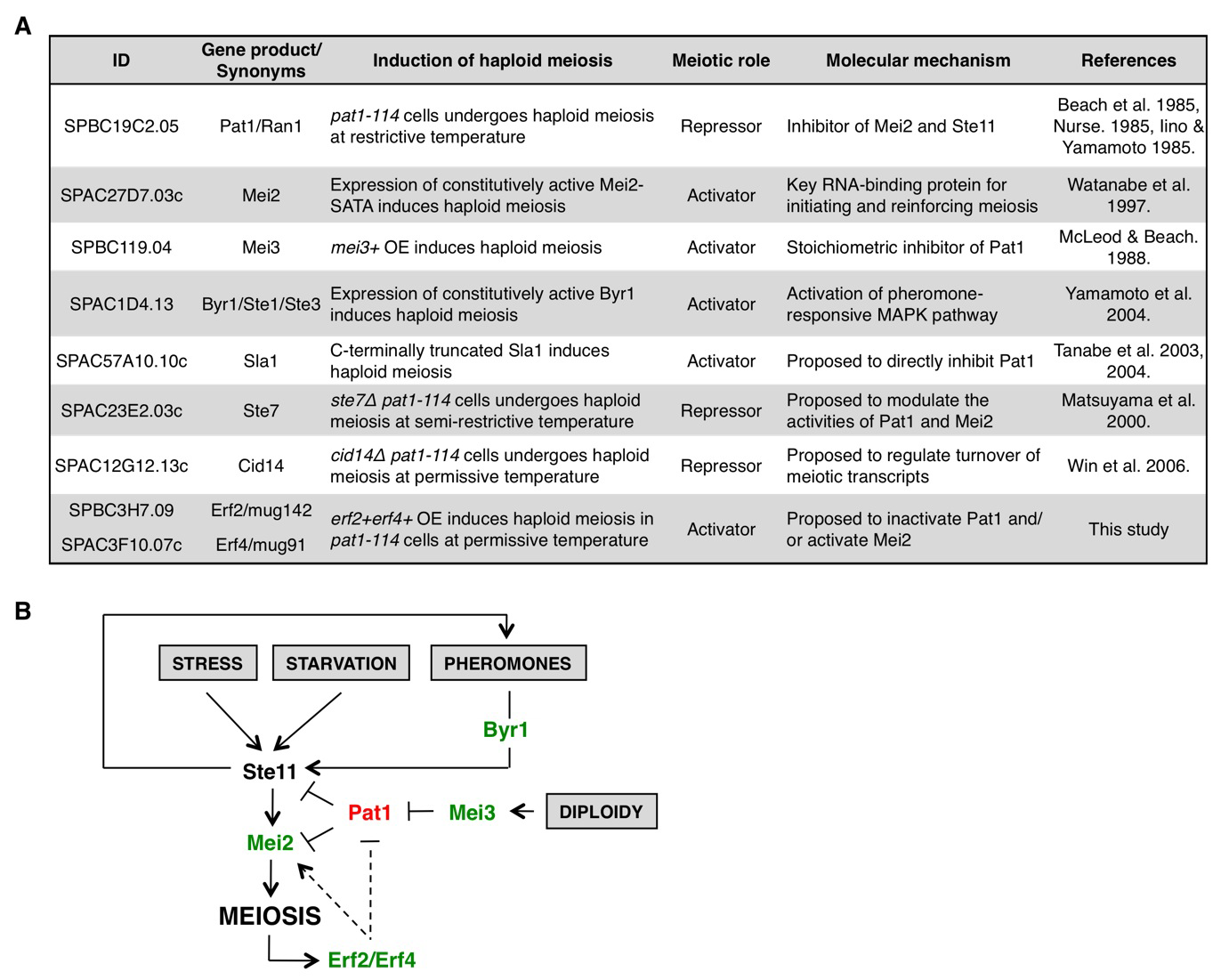

Supplement: Figure S9 — Gene products that induce ectopic meiosis in haploid S. pombe cells. (A) Of the thousands of genes that significantly regulated during meiosis [37],[49], the listed gene products are demonstrated to induce meiosis in haploid cells, highlighting their regulatory roles in the important cellular transition. The exact molecular mechanisms by which some of these gene products modulate meiotic entry remain to be elucidated. Unless otherwise stated, cells are pat1+. (B) Signaling network that regulates fission yeast meiotic entry. In S. pombe, the Mei2 master regulator governs the switch from the mitotic to meiotic cycle. Mei2 integrates extracellular cues (stress, nutrients, and pheromones) primarily through Ste11, and drives meiosis. Mei2 function is tightly regulated by the Pat1 kinase, which inactivates both Mei2 and Ste11, thereby preventing meiotic entry during the mitotic cell cycle [67]. Together, Pat1 and Mei2 constitute the core mitosis-meiosis switch in fission yeast. Under physiological conditions, complete inactivation of Pat1 and meiotic induction strictly requires the stoichiometric inhibitor Mei3, which is only expressed in heterozygous diploids formed after successful conjugation [69]–[71]. Working in concert, these key cellular factors integrate environmental cues and control cellular entry into meiosis. For simplification, many factors and interactions are omitted in this representation. Meiotic activators and repressors are in green and red, respectively. Dashed lines represent proposed mechanism of Erf2–Erf4 in this study. Erf2 and Erf4 expression is significantly upregulated in meiotic cells compared to vegetative cells, and elevated Erf2–Erf4 levels promote the meiotic state by activating Mei2 and/or inactivating Pat1. Dotted lines represent either direct or indirect interactions. (TIF) [file pbio.1001597.s009.tif]
