# Supplementary material for: Quantitative Control of Protein S-Palmitoylation Regulates Meiotic Entry in Fission Yeast
Source: PLoS Biol. 2013 Jul 2;11(7):e1001597. doi: 10.1371/journal.pbio.1001597 (PMC3699447; doi:10.1371/journal.pbio.1001597)
Supplement: Table S1 — S. pombe strains used in this study. (DOC) [file pbio.1001597.s010.doc]

| **Strain** | **Genotype** | **Source** |
| --- | --- | --- |
| PN1 | *h-* | Nurse lab stock |
| Mz53 | *h- erf2Δ::NatMX6* | This study |
| Mz166 | *h- erf4Δ::KanMX6* | This study |
| Mz126 | *h- akr1Δ::KanMX6* | This study |
| Mz120 | *h- pfa5Δ::KanMX6* | This study |
| Mz108 | *h+ erf2Δ::NatMX6 leu1-32::pDUAL(leu1 nmt41:HFY-erf2) ade-M216 ura4-D18* | This study |
| Mz199 | *h+ erf2Δ::NatMX6 leu1-32::pDUAL(leu1 nmt41:HFY-erf2(DHHA)) ade-M216 ura4-D18* | This study |
| Mz471 | *h- pat1-114 nmt1:erf2::KanMX6 rho3-HA_3_::KanMX6* | This study |
| Mz492 | *h- pat1-114 nmt1:erf4::KanMX6 rho3-HA_3_::KanMX6* | This study |
| Mz476 | *h- pat1-114 nmt1:erf2::KanMX6 nmt1:erf4::KanMX6 rho3-HA_3_::KanMX6* | This study |
| Mz504 | *h- pat1-114 nmt41:erf2::KanMX6 nmt41:erf4::KanMX6 rho3-HA_3_::KanMX6* | This study |
| Mz556 | *h- pat1-114 leu1-32::pDUAL(leu1 nmt1:erf2(DHHC))* | This study |
| Mz560 | *h- pat1-114 leu1-32::pDUAL(leu1 nmt1:erf2(DHHA))* | This study |
| Mz518 | *h- nmt1:erf2::KanMX6 nmt1:erf4::KanMX6 rho3-HA_3_::KanMX6* | This study |
| Mz488 | *h- pat1-114 nmt1:erf2::KanMX6 nmt1:erf4::KanMX6 rho3Δ::kanMX6 ura4-D18* | This study |
| Mz587 | *h- pat1-114 nmt1:erf2::KanMX6 nmt1:erf4::KanMX6 ras1Δ::ura4^+^ ura4-D18* | This study |
| Mz5d | *h-/h- pat-114/pat1-114 erf2-HA_3_::KanMX6/erf2-HA_3_::KanMX6* | This study |
| Mz9d | *h-/h- pat-114/pat1-114 erf2Δ::NatMX6/erf2Δ::NatMX6* | This study |
| Mz21d | *h-/h- pat-114/pat1-114 erf4Δ::KanMX6/erf4Δ::KanMX6* | This study |
| Mz25d | *h-/h- pat-114/pat1-114 akr1Δ::KanMX6/akr1Δ::KanMX6* | This study |
| Mz29d | *h-/h- pat-114/pat1-114 pfa5Δ::KanMX6/pfa5Δ::KanMX6* | This study |
| Mz17d | *h-/h- pat-114/pat1-114 akr1Δ::KanMX6/akr1Δ::KanMX6 pfa5Δ::KanMX6/pfa5Δ::KanMX6* | This study |
| Mz60d | *h-/h- pat-114/pat1-114 ras1Δ::KanMX6/ras1Δ::KanMX6* | This study |
| Mz74d | *h-/h- pat-114/pat1-114 isp3Δ::KanMX6/isp3Δ::KanMX6* | This study |
| Mz64d | *h-/h- pat-114/pat1-114 rho3Δ::KanMX6/rho3Δ::KanMX6* | This study |
| Mz88d | *h-/h- pat-114/pat1-114 isp3-HA_3_::KanMX6/isp3-HA_3_::KanMX6* | This study |
| Mz68d | *h-/h- pat-114/pat1-114 rho3-HA_3_::KanMX6/rho3-HA_3_::KanMX6* | This study |
| Mz129d | *h-/h- pat1-114/pat1-114 nmt41:erf2::KanMX6/nmt41:erf2::KanMX6 rho3-HA_3_::KanMX6/rho3-HA_3_::KanMX6* | This study |
| Mz189d | *h-/h- pat1-114/pat1-114 erf2^+^/erf2Δ::ura4^+^ rho3-HA_3_::KanMX6/rho3-HA_3_::KanMX6 ura4-D18/ura4-D18* | This study |
