# Supplementary material for: Quantitative Control of Protein S-Palmitoylation Regulates Meiotic Entry in Fission Yeast
Source: PLoS Biol. 2013 Jul 2;11(7):e1001597. doi: 10.1371/journal.pbio.1001597 (PMC3699447; doi:10.1371/journal.pbio.1001597)
Supplement: Supplementary file 3 [file pbio.1001597.s011.doc]

## Sheet: Sheet1

Identified proteins	Accession Number	Molecular Weight	Vegetative WT		Meiotic WT			Meiotic erf2Δ		Total negative counts	Total positive counts
			DMSO	alk-16	DMSO	alk-16	nett counts	DMSO	alk-16
Isp3	SPAC1F8.05	20 kDa	3	2	30	99	69	8	4	41	105
P-type ATPase	SPAC6C3.06c	117 kDa	0	7	0	14	14	9	9	9	30
Vps13b	SPBC16C6.02c	339 kDa	4	3	0	11	11	4	4	8	18
Rho3	SPAC23C4.08	23 kDa	0	2	0	11	11	0	0	0	13
Sir2	SPBC16D10.07c	53 kDa	4	7	0	8	8	0	0	4	15
Noc1	SPAC4F10.09c	98 kDa	2	4	0	8	8	0	3	2	15
Rad24	SPAC8E11.02c	30 kDa	7	10	0	6	6	0	3	7	19
Cid11	SPBC1685.06	55 kDa	4	6	0	6	6	0	0	4	12
Dcr1	SPCC188.13c	158 kDa	3	0	0	6	6	0	3	3	9
Pre5	SPAC6G10.04c	30 kDa	0	0	0	6	6	0	0	0	6
Gpd1	SPBC215.05	42 kDa	0	2	0	6	6	2	3	2	11
Hxk1	SPAC24H6.04	54 kDa	0	2	0	6	6	0	2	0	10
Sak1	SPAC3G9.14	86 kDa	3	0	0	5	5	0	2	3	7
Ferric-chelate reductase	SPBC947.05c	65 kDa	2	2	0	5	5	2	2	4	9
Ucp8	SPBC83.01	98 kDa	0	0	0	5	5	0	0	0	5
Mss1	SPAC222.05c	55 kDa	0	0	0	5	5	0	0	0	5
Pli1	SPAC1687.05	81 kDa	0	0	0	5	5	0	0	0	5
Non classical export pathway protein	SPBC1685.13	20 kDa	0	0	0	5	5	0	0	0	5
Top1	SPBC1703.14c	94 kDa	0	0	0	5	5	0	2	0	7
Alpha,alpha-trehalose-phosphate synthase	SPACUNK4.16c	107 kDa	0	0	0	5	5	0	2	0	7
UPF0061 family protein	SPAC20G4.05c	64 kDa	0	0	0	5	5	0	2	0	7
Nep1	SPBC17D11.01	47 kDa	0	2	0	5	5	0	0	0	7
Adf1	SPAC20G4.06c	16 kDa	0	2	0	5	5	0	0	0	7
Trk1	SPAC3F10.02c	96 kDa	0	4	4	9	5	2	0	6	13
Rpl14	SPAC1805.13	15 kDa	9	16	0	4	4	0	0	9	20
Uso1	SPAC29E6.03c	122 kDa	4	6	0	4	4	0	0	4	10
Oxa101	SPAC9G1.04	42 kDa	3	0	0	4	4	0	2	3	6
Spt8	SPBC14C8.17c	58 kDa	3	2	0	4	4	0	0	3	6
Hrp3	SPAC3G6.01	159 kDa	3	4	0	4	4	4	6	7	14
Cytoskeletal protein binding protein Sla1 family	SPAC16E8.01	155 kDa	2	0	0	4	4	0	0	2	4
Membrane transporter	SPAC3H1.06c	64 kDa	2	0	0	4	4	0	0	2	4
Ptc4	SPAC4A8.03c	44 kDa	2	2	0	4	4	0	0	2	6
Rhp54	SPAC15A10.03c	97 kDa	2	2	0	4	4	0	2	2	8
Rpl22	SPAC11E3.15	13 kDa	2	2	0	4	4	0	2	2	8
NAD/NADH kinase	SPAC3H5.11	45 kDa	2	3	0	4	4	2	3	4	10
Sir1	SPAC10F6.01c	164 kDa	2	3	0	4	4	0	0	2	7
Vps4	SPAC2G11.06	48 kDa	0	0	0	4	4	2	0	2	4
Klp8	SPAC144.14	57 kDa	0	0	0	4	4	2	0	2	4
Coq3	SPCC162.05	30 kDa	0	0	0	4	4	2	0	2	4
Lhs1	SPAC1F5.06	95 kDa	0	0	0	4	4	2	2	2	6
Rsc7	SPCC1281.05	44 kDa	0	0	0	4	4	0	0	0	4
cdc23	SPBC1347.10	67 kDa	0	0	0	4	4	0	0	0	4
DuF1740 family protein	SPBC20F10.05	113 kDa	0	0	0	4	4	0	0	0	4
Conserved fungal protein	SPCPB16A4.02c	38 kDa	0	0	0	4	4	0	0	0	4
Grx3	SPCC1450.06c	18 kDa	0	0	0	4	4	0	0	0	4
Suc22	SPBC25D12.04	45 kDa	0	0	0	4	4	0	0	0	4
Ght5	SPCC1235.14	60 kDa	0	0	0	4	4	0	0	0	4
FAD-dependent amino acid oxidase	SPAC6G10.06	42 kDa	0	0	0	4	4	0	0	0	4
Rrp6	SPAC1F3.01	90 kDa	0	0	0	4	4	0	0	0	4
Htb1	SPCC622.09	14 kDa	0	0	0	4	4	0	0	0	4
zf-CCHC type zinc finger protein	SPAC683.02c	25 kDa	0	0	0	4	4	0	0	0	4
Ptc2	SPCC1223.11	41 kDa	0	0	0	4	4	0	0	0	4
ATP-dependent RNA helicase, eIF4A related	SPAC1F5.10	45 kDa	0	0	0	4	4	0	0	0	4
Rds1	SPAC343.12	44 kDa	0	0	0	4	4	0	0	0	4
Shf1	SPAC22F8.12c	19 kDa	0	0	0	4	4	0	2	0	6
Aif1	SPAC26F1.14c	62 kDa	0	0	0	4	4	0	2	0	6
Ppp1	SPBC19F5.05c	69 kDa	0	0	0	4	4	0	2	0	6
Mrpl32	SPBC1604.13c	12 kDa	0	0	0	4	4	0	2	0	6
Rpn2	SPBC17D11.07c	107 kDa	0	2	0	4	4	4	2	4	8
Ubp22	SPCC188.08c	129 kDa	0	2	0	4	4	4	3	4	9
Pga2	SPBC27.01c	16 kDa	0	2	0	4	4	3	2	3	8
Arh1	SPBC3B8.01c	53 kDa	0	2	0	4	4	3	3	3	9
Spt5	SPAC23C4.19	109 kDa	0	2	0	4	4	2	0	2	6
Conserved eukaryotic protein	SPAC1687.04	57 kDa	0	2	0	4	4	0	0	0	6
Sequence orphan	SPBC405.02c	50 kDa	0	2	0	4	4	0	0	0	6
Str2	SPCC61.01c	66 kDa	0	2	0	4	4	0	2	0	8
transcription factor, zf-fungal binuclear cluster type	SPAC1399.05c	61 kDa	3	3	0	3	3	0	0	3	6
RNA binding protein	SPAC4G8.03c	88 kDa	2	2	0	3	3	0	0	2	5
Dbp9	SPCC1494.06c	67 kDa	2	2	0	3	3	0	0	2	5
guanyl-nucleotide exchange factor	SPAC11E3.11c	106 kDa	0	0	0	3	3	3	3	3	6
Mcm3	SPCC1682.02c	97 kDa	0	0	0	3	3	3	4	3	7
Pig-F	SPCC1450.15	57 kDa	0	0	0	3	3	2	2	2	5
Ubiquitin-protein ligase E3	SPBC947.10	77 kDa	0	0	0	3	3	2	2	2	5
Yop1	SPCC830.08c	21 kDa	0	0	0	3	3	2	2	2	5
Sequence orphan	SPAC821.03c	54 kDa	0	0	0	3	3	2	3	2	6
Sequence orphan	SPAC13G7.09c	16 kDa	0	0	0	3	3	0	0	0	3
AAA family ATPase, unknown biological role	SPBC947.01	72 kDa	0	0	0	3	3	0	0	0	3
Sec6	SPCC1235.10c	83 kDa	0	0	0	3	3	0	0	0	3
Ulp1	SPBC19G7.09	65 kDa	0	0	0	3	3	0	0	0	3
Apl4	SPCP1E11.06	96 kDa	0	0	0	3	3	0	0	0	3
D123 family	SPAP27G11.03	37 kDa	0	0	0	3	3	0	0	0	3
Hst4	SPAC1783.04c	47 kDa	0	0	0	3	3	0	0	0	3
Pinin homolog	SPAC26F1.02	23 kDa	0	0	0	3	3	0	0	0	3
CorA family magnesium ion transporter	SPAC17A2.14	71 kDa	0	0	0	3	3	0	0	0	3
Aspartate kinase	SPBC19F5.04	57 kDa	0	0	0	3	3	0	0	0	3
Fumarate lyase superfamily	SPBC8E4.05c	50 kDa	0	0	0	3	3	0	0	0	3
Transcription factor	SPAPB1A11.04c	79 kDa	0	0	0	3	3	0	0	0	3
tRNA nucleotidyltransferase	SPAC1093.04c	58 kDa	0	0	0	3	3	0	0	0	3
WD repeat protein, human WDR55 family	SPAC1A6.02	40 kDa	0	0	0	3	3	0	0	0	3
Biotin-protein ligase	SPBC30D10.07c	71 kDa	0	0	0	3	3	0	0	0	3
Transcription factor	SPBC19C7.10	48 kDa	0	0	0	3	3	0	0	0	3
Ctf1	SPBC3B9.11c	40 kDa	0	0	0	3	3	0	0	0	3
Sec15	SPCC1183.01	91 kDa	0	0	0	3	3	0	0	0	3
Nak1	SPBC17F3.02	71 kDa	0	0	0	3	3	0	0	0	3
Rpt6	SPBC23G7.12c	45 kDa	0	0	0	3	3	0	0	0	3
Nucleoporin Nup157/170	SPAC890.06	148 kDa	0	0	0	3	3	0	0	0	3
Mitochondrial citrate transporter	SPAC19G12.05	32 kDa	0	0	0	3	3	0	0	0	3
Amidase	SPAC869.01	65 kDa	0	0	0	3	3	0	0	0	3
Uve1	SPBC19C7.09c	69 kDa	0	0	0	3	3	0	0	0	3
Leu3	SPBC3E7.16c	64 kDa	0	0	0	3	3	0	0	0	3
Adaptin	SPAC1F3.05	57 kDa	0	0	0	3	3	0	0	0	3
Ubp6	SPAC6G9.08	52 kDa	0	0	0	3	3	0	0	0	3
ZIP zinc transporter 1	SPAP8A3.03	50 kDa	0	0	0	3	3	0	0	0	3
Taf1	SPAC1002.04c	23 kDa	0	0	0	3	3	0	0	0	3
Phx1	SPAC32A11.03c	104 kDa	0	0	0	3	3	0	0	0	3
Pex7	SPAC17D4.01	35 kDa	0	0	0	3	3	0	0	0	3
Gln1	SPAC23H4.06	40 kDa	0	0	0	3	3	0	0	0	3
Ptr6	SPAC13F5.02c	45 kDa	0	0	0	3	3	0	0	0	3
Ubi1	SPAC11G7.04	15 kDa	0	0	0	3	3	0	0	0	3
Las1-like protein	SPBC16C6.12c	54 kDa	0	0	0	3	3	0	0	0	3
Gpi18	SPAC18B11.05	49 kDa	0	0	0	3	3	0	0	0	3
AMP-activated protein kinase beta subunit	SPCC1919.03c	33 kDa	0	0	0	3	3	0	0	0	3
Cbs2	SPAC1556.08c	37 kDa	0	0	0	3	3	0	0	0	3
Sdh3	SPCC330.12c	20 kDa	0	0	0	3	3	0	0	0	3
DUF726 family protein	SPAC607.08c	64 kDa	0	0	3	6	3	0	3	3	9
Sti1	SPCC645.14c	66 kDa	0	2	2	5	3	3	3	5	10
Pmc2	SPAC2F7.04	51 kDa	3	4	0	2	2	0	0	3	6
Rps21	SPBC18E5.06	10 kDa	3	5	0	2	2	0	0	3	7
Lsk1	SPAC2F3.15	67 kDa	2	2	0	2	2	0	0	2	4
Swi2	SPAC1142.03c	82 kDa	2	2	0	2	2	0	0	2	4
Dbp3	SPBC17D1.06	64 kDa	2	2	0	2	2	0	0	2	4
PQ loop protein	SPAC2E12.03c	32 kDa	2	3	0	2	2	3	5	5	10
Ypt3	SPAC18G6.03	24 kDa	2	3	0	2	2	0	0	2	5
TRP-like ion channel	SPCC663.14c	77 kDa	0	0	0	2	2	2	2	2	4
Irs1	SPBC8D2.06	123 kDa	0	0	0	2	2	2	3	2	5
Lsd90	SPBC16E9.16c	82 kDa	0	0	0	2	2	2	3	2	5
Pdt1	SPAC27F1.08	58 kDa	0	0	0	2	2	2	3	2	5
alpha-1,2-mannosyltransferase	SPBC16H5.09c	44 kDa	0	0	0	2	2	2	3	2	5
Rec11	SPCC4E9.01c	107 kDa	0	0	0	2	2	0	0	0	2
Conserved fungal protein	SPCC1494.08c	31 kDa	0	0	0	2	2	0	0	0	2
Nat10	SPAC20G8.09c	116 kDa	0	0	0	2	2	0	0	0	2
Abp2	SPBC1861.02	60 kDa	0	0	0	2	2	0	0	0	2
Sid4	SPBC244.01c	74 kDa	0	0	0	2	2	0	0	0	2
Aminotransferase class-III, unknown specificty	SPAC27F1.05c	53 kDa	0	0	0	2	2	0	0	0	2
Mfh1	SPAC9.05	97 kDa	0	0	0	2	2	0	0	0	2
Conserved fungal protein	SPAC2F3.14c	38 kDa	0	0	0	2	2	0	0	0	2
Glc9	SPAC17A5.09c	35 kDa	0	0	0	2	2	0	0	0	2
Sequence orphan	SPBC2G2.14	60 kDa	0	0	0	2	2	0	0	0	2
Atp23	SPCC320.12	22 kDa	0	0	0	2	2	0	0	0	2
Spermidine family transporter	SPBC36.03c	59 kDa	0	0	0	2	2	0	0	0	2
Mug100	SPBC16E9.07	36 kDa	0	0	0	2	2	0	0	0	2
DUF1716 family protein	SPAC1952.06c	65 kDa	0	0	0	2	2	0	0	0	2
Ltv1	SPAC3F10.17	44 kDa	0	0	0	2	2	0	0	0	2
Arg7	SPBC1773.14	52 kDa	0	0	0	2	2	0	0	0	2
Cmk1	SPACUNK12.02c	38 kDa	0	0	0	2	2	0	0	0	2
Mitochondrial tRNA	SPAC12B10.08c	52 kDa	0	0	0	2	2	0	0	0	2
Ran GTP-binding protein	SPAC31A2.10	51 kDa	0	0	0	2	2	0	0	0	2
Acetyl-CoA hydrolase	SPAC1952.09c	58 kDa	0	0	0	2	2	0	0	0	2
C2 domain protein	SPCC962.01	156 kDa	0	0	0	2	2	0	0	0	2
Transcription factor	SPAC105.03c	81 kDa	0	0	0	2	2	0	0	0	2
Arrestin/ PY protein 1	SPAC31A2.12	66 kDa	0	0	0	2	2	0	0	0	2
Tht1	SPAC13C5.03	63 kDa	0	0	0	2	2	0	0	0	2
Sbh1	SPBC2G2.03c	10 kDa	0	0	0	2	2	0	0	0	2
Nap1	SPCC364.06	44 kDa	0	0	0	2	2	0	0	0	2
Sequence orphan	SPAC7D4.13c	36 kDa	0	0	0	2	2	0	0	0	2
Ark1	SPCC320.13c	41 kDa	0	0	0	2	2	0	0	0	2
Svf1	SPCC584.11c	43 kDa	0	0	0	2	2	0	0	0	2
LEA domain protein	SPAC23C4.05c	50 kDa	0	0	0	2	2	0	0	0	2
Short chain dehydrogenase	SPAC19A8.06	45 kDa	0	0	0	2	2	0	0	0	2
TLDc domain protein 2	SPBC21.02	59 kDa	0	0	0	2	2	0	0	0	2
NADPH-dependent diflavin oxidoreductase	SPAC1296.06	64 kDa	0	0	0	2	2	0	0	0	2
Gma12	SPCC736.04c	43 kDa	0	0	0	2	2	0	0	0	2
Rsv2	SPBC1105.14	69 kDa	0	0	0	2	2	0	0	0	2
Pgp1	SPCC1259.10	46 kDa	0	0	0	2	2	0	0	0	2
Mep33	SPBC28F2.02	33 kDa	0	0	0	2	2	0	0	0	2
Rft1	SPBC887.19	60 kDa	0	0	0	2	2	0	0	0	2
Mitochondrial m-AAA protease	SPBC543.09	85 kDa	0	0	0	2	2	0	0	0	2
Hta2	SPAC19G12.06c	14 kDa	0	0	0	2	2	0	0	0	2
Rae1	SPBC16A3.05c	39 kDa	0	0	0	2	2	0	0	0	2
Hhp1	SPBC3H7.15	42 kDa	0	0	0	2	2	0	0	0	2
Wee1	SPCC18B5.03	96 kDa	0	0	0	2	2	0	0	0	2
Pfs2	SPAC12G12.14c	58 kDa	0	0	0	2	2	0	0	0	2
Cytoskeletal signaling protein	SPAC637.13c	56 kDa	0	0	0	2	2	0	0	0	2
Mcp2	SPCC1682.08c	79 kDa	0	0	0	2	2	0	0	0	2
Mug73	SPCC31H12.02c	35 kDa	0	0	0	2	2	0	0	0	2
Mbx1	SPBC19G7.06	51 kDa	0	0	0	2	2	0	0	0	2
Apc11	SPAC343.03	11 kDa	0	0	0	2	2	0	0	0	2
Nudix family hydrolase	SPAC14C4.10c	38 kDa	0	0	0	2	2	0	0	0	2
DUF544 family protein	SPAC12G12.11c	42 kDa	0	0	0	2	2	0	0	0	2
Ppk29	SPBC557.04	96 kDa	0	0	0	2	2	0	0	0	2
Rfc2	SPAC23D3.02	38 kDa	0	0	0	2	2	0	0	0	2
Sgf29	SPBC1921.07c	28 kDa	0	0	0	2	2	0	0	0	2
Nicotinic acid plasma membrane transporter	SPAC1002.16c	55 kDa	0	0	0	2	2	0	0	0	2
Peptidase	SPCC1259.02c	92 kDa	0	0	0	2	2	0	0	0	2
Steriod dehydrogenase	SPAC7D4.09c	32 kDa	0	0	0	2	2	0	0	0	2
Rhp18	SPBC1734.06	43 kDa	0	0	0	2	2	0	0	0	2
Membrane transporter	SPCC965.13	60 kDa	0	0	0	2	2	0	0	0	2
RINT1 family protein	SPBC691.02c	79 kDa	0	0	0	2	2	0	0	0	2
Rad17	SPAC14C4.13	69 kDa	0	0	0	2	2	0	0	0	2
Ura3	SPAC57A10.12c	48 kDa	0	0	0	2	2	0	0	0	2
Fun1	SPCC18.18c	56 kDa	0	0	0	2	2	0	0	0	2
Erv41	SPBC2G5.04c	38 kDa	0	0	0	2	2	0	0	0	2
Sce3	SPBC18H10.04c	43 kDa	0	0	0	2	2	0	0	0	2
IMP 5'-nucleotidase	SPBC30D10.03c	46 kDa	0	0	0	2	2	0	0	0	2
Rax2	SPAC6F6.06c	128 kDa	0	0	0	2	2	0	0	0	2
CTNS domain protein	SPAC4C5.03	34 kDa	0	0	0	2	2	0	0	0	2
Nup120	SPBC3B9.16c	130 kDa	0	0	0	2	2	0	0	0	2
Asa1	SPAC1006.02	41 kDa	0	0	0	2	2	0	0	0	2
Sequence orphan	SPAC24C9.04	14 kDa	0	0	0	2	2	0	0	0	2
Spp2	SPBC17D11.06	53 kDa	0	0	0	2	2	0	0	0	2
Clr2	SPAC1B3.17	62 kDa	0	0	0	2	2	0	0	0	2
Tea1	SPCC1223.06	127 kDa	0	0	0	2	2	0	0	0	2
Sequence orphan	SPAC17A2.07c	20 kDa	0	0	0	2	2	0	0	0	2
Nab2	SPAC14C4.06c	34 kDa	0	0	0	2	2	0	0	0	2
Conserved protein	SPAC6G9.01c	10 kDa	0	0	0	2	2	0	0	0	2
Ubiquitin-protein ligase E3	SPBC16G5.03	31 kDa	0	0	0	2	2	0	0	0	2
Atp6	SPMIT.07	28 kDa	0	0	0	2	2	0	0	0	2
Histone acetyltransferase complex subunit	SPAC25H1.06	47 kDa	0	0	0	2	2	0	0	0	2
SRR1 family protein	SPBC14C8.13	29 kDa	0	0	0	2	2	0	0	0	2
Sgf11	SPAC57A10.14	13 kDa	0	0	0	2	2	0	0	0	2
Atf31	SPAC22F3.02	24 kDa	0	0	0	2	2	0	0	0	2
Swd1	SPAC23H3.05c	45 kDa	0	0	0	2	2	0	0	0	2
Short chain dehydrogenase	SPAC977.08	26 kDa	0	0	0	2	2	0	0	0	2
Eng2	SPAC23D3.10c	78 kDa	0	0	0	2	2	0	0	0	2
Swd1	SPBC354.02c	53 kDa	0	0	0	2	2	0	0	0	2
Mis16	SPCC1672.10	48 kDa	0	0	0	2	2	0	0	0	2
Membrane transporter	SPCC417.10	57 kDa	0	0	0	2	2	0	0	0	2
Psy1	SPCC825.03c	33 kDa	0	0	0	2	2	0	0	0	2
Moc3	SPAC821.07c	55 kDa	0	0	0	2	2	0	0	0	2
RNA-binding protein, G-patch type	SPAC2G11.04	33 kDa	0	0	0	2	2	0	0	0	2
Cwf20	SPCC4B3.14	33 kDa	0	0	0	2	2	0	0	0	2
Mug117	SPCC645.11c	22 kDa	0	0	0	2	2	0	0	0	2
DUF1000 family protein	SPBP35G2.02	23 kDa	0	0	0	2	2	0	0	0	2
Mitochondrial ribosomal protein subunit L9	SPCC777.17c	12 kDa	0	0	0	2	2	0	0	0	2
ORMDL family protein	SPBC119.09c	21 kDa	0	0	0	2	2	0	0	0	2
Tif212	SPAC32A11.04c	36 kDa	0	0	0	2	2	0	0	0	2
Mdm10	SPAC17H9.17c	42 kDa	0	0	0	2	2	0	0	0	2
tRNA specific adenosine deaminase	SPBC16A3.06	44 kDa	0	0	0	2	2	0	0	0	2
Hem2	SPAC1805.06c	36 kDa	0	0	0	2	2	0	0	0	2
Imidazoleglycerol-phosphate synthase	SPAC222.08c	26 kDa	0	0	0	2	2	0	0	0	2
Diacylglycerol cholinephosphotranferase/ diacylglycerol ethanolaminesphotranferase	SPAC22A12.10	44 kDa	0	0	0	2	2	0	0	0	2
Metal dependent phosphohydrolase	SPCC4G3.17	23 kDa	0	0	0	2	2	0	0	0	2
Gpi12	SPAPB2B4.01c	28 kDa	0	0	0	2	2	0	0	0	2
tspO homolog	SPBC725.10	18 kDa	0	0	0	2	2	0	0	0	2
Btn1	SPAC607.09c	44 kDa	0	0	0	2	2	0	0	0	2
Sequence orphan	SPBPB21E7.05	15 kDa	0	0	0	2	2	0	0	0	2
Amino acid permease, unknown 15	SPCC74.04	60 kDa	0	0	0	2	2	0	0	0	2
Rng3	SPCC613.04c	84 kDa	0	0	2	4	2	0	0	2	4
