# Supplementary material for: Quantitative Control of Protein S-Palmitoylation Regulates Meiotic Entry in Fission Yeast
Source: PLoS Biol. 2013 Jul 2;11(7):e1001597. doi: 10.1371/journal.pbio.1001597 (PMC3699447; doi:10.1371/journal.pbio.1001597)
Supplement: Supplementary file 4 [file pbio.1001597.s012.doc]

## Sheet: Sheet1

Gene	ID	Left	Right
actin	SPBC32H8.12c	CGAACGTGAAATTGTTCGTG	GGAGGAAGATTGAGCAGCAG
erf2	SPBC3H7.09 	CTTTTTGGCTCTGGCATCAT	AATTCCTGGATCAGCAGTCG
erf4	SPAC3F10.07c	CGCAATATGGGGTTTTTGAG	GGCAGAGGGAAGTGTTCGTA
pfa3	SPBC2F12.15c	TGCCATCACCAAAACTTTCA	TCATTCCAACAGCAAAAGCA
pfa5	SPBC691.01	GGTGATTTACCATGGGATCG	GAAGCGGCAAAATCCAATTA
swf1	SPBC13G1.07	TCCATACTCCGCCTTGGTTA	CCCAGTTCCCCTTCACTTTT
akr1	SPAC2F7.10	TTAGCCGCTAGTCAGGGAGA	AATGCAGTTGCTCCACCTTC
